# Pre-eclampsia is associated with a twofold increase in diabetes: a systematic review and meta-analysis

**DOI:** 10.1007/s00125-016-4098-x

**Published:** 2016-09-19

**Authors:** Pensee Wu, Chun Shing Kwok, Randula Haththotuwa, Rafail A. Kotronias, Aswin Babu, Anthony A. Fryer, Phyo K. Myint, Carolyn A. Chew-Graham, Mamas A. Mamas

**Affiliations:** 1 Institute for Science and Technology in Medicine, Keele University, Guy Hilton Research Centre, Thornburrow Drive, Hartshill, Stoke-on-Trent, ST4 7QB UK; 2 Academic Obstetrics and Gynaecology, Maternity Centre, Royal Stoke University Hospital, Stoke-on-Trent, UK; 3 Keele Cardiovascular Research Group, Keele University, Stoke-on-Trent, UK; 4 The Heart Centre, Royal Stoke University Hospital, Stoke-on-Trent, UK; 5 Primary Care and Health Sciences, Keele University, Stoke-on-Trent, UK; 6 Epidemiology Group, Institute of Applied Health Sciences, University of Aberdeen, Aberdeen, UK; 7 NIHR Collaboration for Leadership in Applied Health Research and Care (CLAHRC) West Midlands, Keele University, Stoke-on-Trent, UK

**Keywords:** Diabetes mellitus, Meta-analysis, Pre-eclampsia, Risk predictors, Screening, Systematic review

## Abstract

**Aims/hypothesis:**

Pre-eclampsia is a pregnancy-specific multisystem disorder and a state of physiological insulin resistance. Our aim was to systematically evaluate and quantify the evidence on the relationship between pre-eclampsia and the future risk of diabetes.

**Methods:**

We conducted a systematic review and meta-analysis of studies that evaluated diabetes in women with and without pre-eclampsia. We performed a systematic search of MEDLINE and EMBASE to identify relevant studies. Independent double data extractions were conducted by four reviewers. Random-effects meta-analysis was used to estimate the risk of future diabetes following pre-eclampsia.

**Results:**

A total of 21 studies were identified with more than 2.8 million women, including more than 72,500 women with pre-eclampsia. Meta-analysis of studies that adjusted for potential confounders demonstrated that pre-eclampsia was independently associated with an increased risk of future diabetes (RR 2.37 [95% CI 1.89, 2.97]). This risk appeared in studies that followed women from less than 1 year postpartum (RR 1.97 [95% CI 1.35, 2.87]) and persisted to more than 10 years postpartum (RR 1.95 [95% CI 1.28, 2.97]). After adjusting for BMI or gestational diabetes, pre-eclampsia remained linked with an increased risk of future diabetes (RR 2.38 [95% CI 1.74, 3.24] and RR 2.36 [95% CI 1.94, 2.88], respectively).

**Conclusions/interpretation:**

Pre-eclampsia is independently associated with a twofold increase in future diabetes. Our study highlights the importance of clinical risk assessment for the future development of diabetes in women with pre-eclampsia. We recommend detailed evaluation of a screening programme for diabetes in this high-risk population.

**Electronic supplementary material:**

The online version of this article (doi:10.1007/s00125-016-4098-x) contains peer-reviewed but unedited supplementary material, which is available to authorised users.

## Introduction

Pre-eclampsia is a pregnancy-specific multisystem disorder that affects 5–8% of pregnancies [1]. It frequently manifests as new-onset hypertension and proteinuria. It is the most common cause of severe perinatal morbidity and is responsible for more than 50,000 maternal deaths per annum globally [2]. Pregnancy is known to be a state of physiological insulin resistance and relative glucose intolerance [3]. Insults to the cardiovascular and renal systems from pre-eclampsia often persist postnatally, with insulin resistance [4], diffuse vascular endothelial dysfunction [5] and inflammatory factor activation [6] reported, although it is unclear whether these are pre-existing conditions prior to the pregnancy or longer-term sequelae of pre-eclampsia. Many of these pathophysiological mechanisms are also linked to the future development of diabetes [7]. Furthermore, lower insulin sensitivity and higher insulin levels have been found in women with a previous history of pre-eclampsia [8].

It remains controversial as to whether pre-eclampsia has long-term metabolic sequelae and is an independent risk factor for the future development of diabetes, as it is difficult to separate pre-eclampsia from confounding factors that are associated with future incident diabetes. The existing literature provides conflicting data, with some studies showing significant increases in the risk of future diabetes [9, 10] and others not observing such a relationship [11, 12]. Many of the studies that have focussed on the association between pre-eclampsia and future incident diabetes have reported limited clinical details for the cohorts studied, and have not adjusted for BMI [9], family history of diabetes [10] or other factors that are known to increase the future risk of incident diabetes, hence raising the potential for unmeasured or unreported confounders contributing to the associations reported. This systematic review and meta-analysis aimed to quantify the risk of diabetes in later life following pre-eclampsia in pregnancy. Here, we provide an overview of the relevant studies and of the association between pre-eclampsia and future incident diabetes.

## Methods

### Eligibility criteria

We selected studies that evaluated diabetes in women with and without pre-eclampsia. Diabetes could be type 1, type 2, any diabetes or the use of diabetes medications such as insulin or oral antidiabetic agents. There were no restrictions on the definition of pre-eclampsia. Included studies had to have at least two groups (one with and one without pre-eclampsia) and to provide results that allowed risk estimates to be calculated. Studies were included if they evaluated some form of risk or odds (e.g. RR, HR, OR) that measured the association with diabetes in patients with or without pre-eclampsia, or reported crude results that enabled calculation of an RR. Crude results that met these criteria had to evaluate diabetes patients or total participants with pre-eclampsia, or diabetes patients or total participants without pre-eclampsia. We planned to contact authors to clarify results where the data reported were uncertain, but all of the studies that met the inclusion criteria had clear reporting of results. There were no restrictions on study design or cohort type. We excluded publications that were not published in the English language.

### Data sources and searches

We searched MEDLINE and EMBASE using Ovid SP for studies from 2005 to August 2015 (see electronic supplementary material [ESM] Methods for comprehensive search terms). This is because the diagnostic criteria for both pre-eclampsia [13] and diabetes [14] were changed in 2001 and 2006, respectively. The relevant primary studies for inclusion in this analysis were drawn from a comprehensive programme of evidence synthesis that explored the association between pre-eclampsia and adverse cardiovascular or metabolic outcomes. We also examined the reference lists of relevant studies and reviews for additional studies that might meet the inclusion criteria.

### Study selection and data extraction

Four reviewers (PW, RH, RAK, AB) screened all of the titles and abstracts retrieved from the search to identify studies that met the inclusion criteria. The full manuscripts of studies that potentially met the inclusion criteria were reviewed, and the final decision to include or exclude studies was made with two other reviewers (CSK, MAM). Independent double extractions were performed by four reviewers (PW, RH, RAK, AB), and data were collected on study design, year, country, number of women, mean age, parity, cohort characteristics, definition of pre-eclampsia, outcomes assessed, timing of assessment and results.

### Study quality assessment

We assessed the quality of the studies using the Newcastle–Ottawa Scale [15]. The representativeness of the exposed cohort was based on whether the study only evaluated patients of a subgroup of the general population, thus limiting generalisability compared with the general female population. Selection of the non-exposed cohort was considered by evaluating whether members of reference or comparator group without pre-eclampsia were included based on a specific criteria, or were a non-selected group. Ascertainment of exposure was evaluated by considering the likelihood that cases were misclassified as having pre-eclampsia when they did not, or that cases were wrongly classified as not having pre-eclampsia. The methods for studies where all patients were assessed for pre-eclampsia were deemed to be more reliable. Studies that excluded patients with baseline diabetes were considered to be more reliable in terms of demonstrating that the outcome of interest was not present at the start of the study. The comparability of the cohort was considered by whether the study had baseline differences between the groups with and without pre-eclampsia, and whether the analysis matched or adjusted for these differences. Higher quality studies either did not have differences in baseline characteristics or adjusted for differences in these characteristics. Assessment of outcome was considered a quality criterion, where the highest quality was an independent blind assessment, followed by record linkage. Outcome assessments were considered to be low quality if there were self-reported results or no description of the results. Duration of follow-up was another quality indicator where a study was deemed high quality if it followed up patients for more than 10 years. The final quality assessment area was the adequacy of follow-up, which was deemed to be high if all participants were accounted for and followed, with loss to follow-up of less than 10%. Studies were considered to be low quality in this area if loss to follow-up was more than 10% or if no statement regarding follow-up was provided. We planned to conduct asymmetry testing for publication bias provided there were more than ten studies in the meta-analysis and statistical heterogeneity was less than 50% [16].

### Data synthesis and analysis

We used Review Manager (RevMan) [Computer program] version 5.3.5 (Copenhagen: The Nordic Cochrane Centre, The Cochrane Collaboration, 2014) to conduct random-effects meta-analysis using the inverse variance method for pooling log RRs. We used random effects because the studies were conducted in a wide range of settings in different populations, making it necessary to take heterogeneity into account for the pooled effect estimate. Where possible, we chose to pool adjusted risk estimates from primary studies; where these data were not available, raw data were used to calculate unadjusted risk estimates. The primary outcome was any diabetes and the analysis was performed considering adjusted and unadjusted groups separately. Secondary analysis was performed considering the risk of types 1 and 2 diabetes separately. Statistical heterogeneity was assessed using the I^2^ statistic, where I^2^ values of 30–60% represented a moderate level of heterogeneity [17]. Where there was moderate or a greater degree of heterogeneity, we performed leave-one-out analysis to identify studies that contributed to a high degree of heterogeneity. Sensitivity analysis was performed considering the follow-up of the studies for diabetes (<1, 1–5, 6–10 and >10 years), exclusion of women with baseline diabetes and hypertension, and baseline differences in BMI, age and gestational diabetes. For the sensitivity analysis on gestational diabetes, in cases where there were more than two separate groups being studied, we selected the group with no pre-eclampsia vs the group with pre-eclampsia but no gestational diabetes for data abstraction.

## Results

### Description of studies included in analysis

**Fig. 1:**
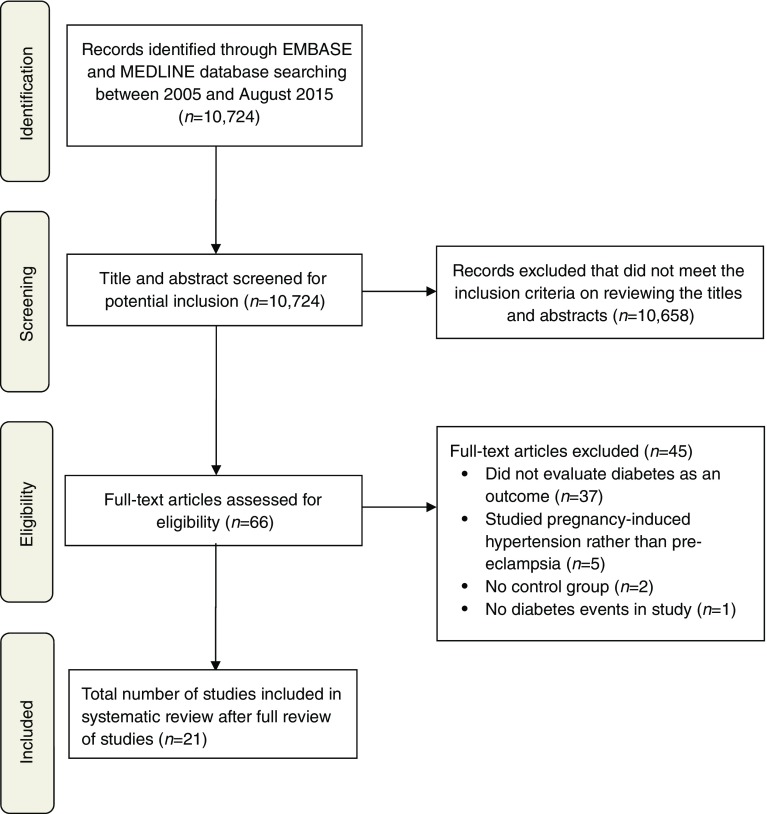
Flow diagram of study inclusion (following PRISMA 2009 recommendations)

The process of study selection is shown in Fig. 1. Out of 10,724 titles and abstracts screened, there were 21 relevant studies including a total of 2,883,658 women (range 140–948,035 women in each study). The study designs and participant demographics are shown in ESM Table 1. There were 72,860 women with pre-eclampsia and 1,961,159 women without pre-eclampsia from 20 studies that reported the numbers of women in each group. Four studies were of primiparous women [18–21] and 17 were of women of any parity [9–12, 18, 22–33]. The mean age of the women ranged from 23.4 to 31 years at index pregnancy.

### Quality assessment in included studies

The quality assessment of the included studies using the Newcastle–Ottawa Scale [15] is shown in ESM Table 2 and 3. A total of 19 studies were deemed to have reliable methods for ascertaining pre-eclampsia, which included review of medical charts, discharge codes, national databases and other registries [9, 10, 12, 18–26, 28–34], while 18 used reliable methods for ascertaining diabetes, which included blood glucose testing, medical records, direct assessment and use of insurance or registry data [9, 10, 12, 18–28, 30, 32–34]. Loss to follow-up was less than 10% in ten studies [9–11, 19, 22, 26, 28, 30, 32, 34]. An adjusted analysis was used in 16 studies [9, 10, 18–25, 27–29, 32–34].

### Determining pre-eclampsia and study results

A variety of different methods were used to ascertain pre-eclampsia. The most common definition used was the 2014 definition of the International Society of the Studies of Hypertension in Pregnancy [35]. Follow-up for incident diabetes was up to 46 years. Results are shown in ESM Table 4.

### Pooled analysis of pre-eclampsia and diabetes

**Fig. 2:**
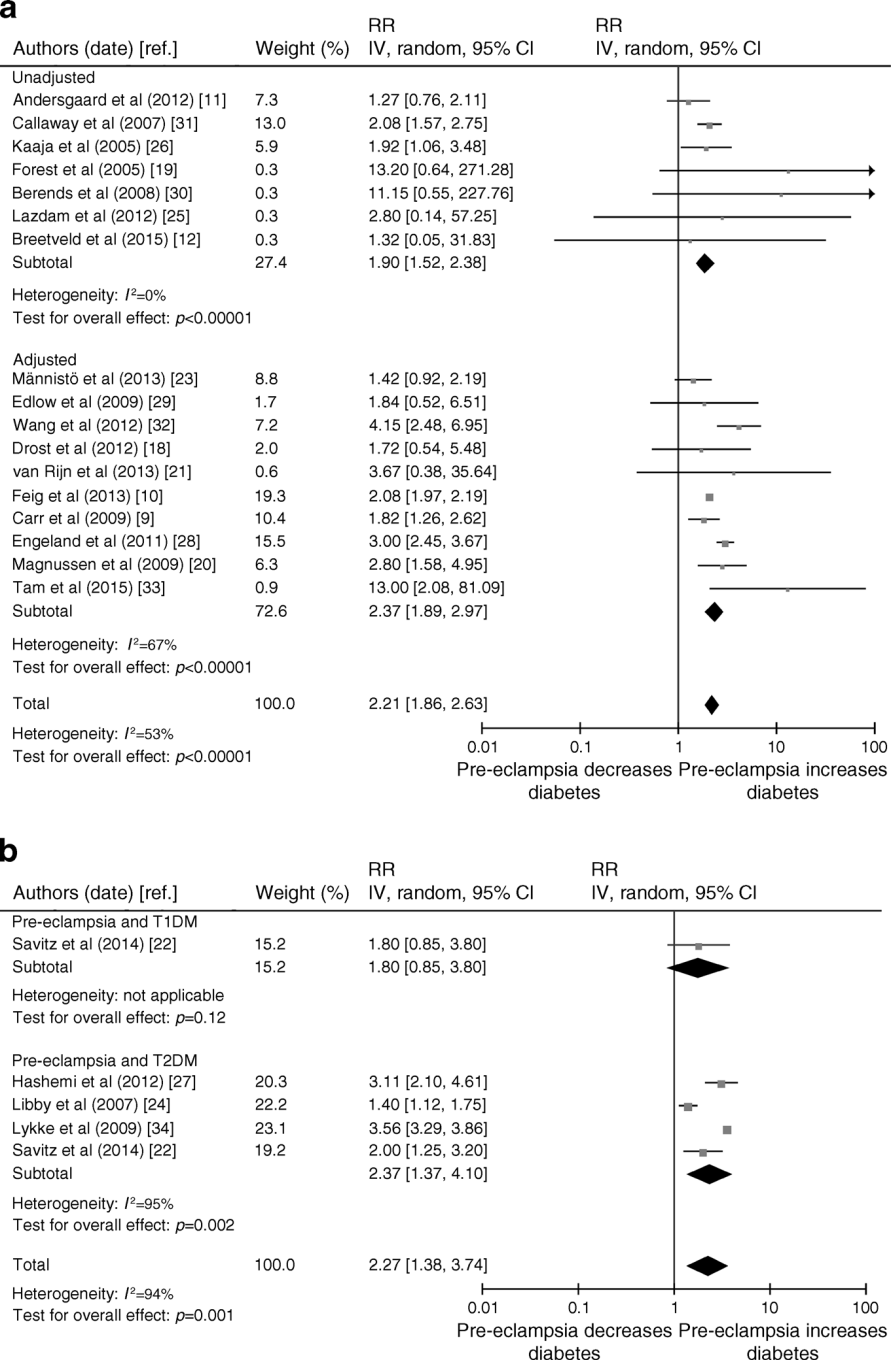
Risk of diabetes with pre-eclampsia. (**a**) Risk of any diabetes with pre-eclampsia, unadjusted and adjusted for confounders. (**b**) Risk of types 1 and 2 diabetes with pre-eclampsia. Risk estimates may not exactly match those reported in ESM Table 4 because of rounding differences and asymmetry in 95% CIs in the original studies. IV, inverse variance; T1DM, type 1 diabetes; T2DM, type 2 diabetes

A total of 17 studies looked at the future risk of any diabetes (type 1 or 2) in association with pre-eclampsia, ten of which adjusted for potential confounders (Fig. 2a) [9, 10, 18, 20, 21, 23, 28, 29, 32, 33]. The confounders that were adjusted for in the analyses are presented in ESM Table 3. The pooled results suggested a significant increase in future incident diabetes risk associated with pre-eclampsia (RR 2.21 [95% CI 1.86, 2.63], *I* ^2^ = 53%) (Fig. 2a). The results were also statistically significant for the studies that adjusted for baseline confounders (adjusted [a]RR 2.37 [95% CI 1.89, 2.97] *I* ^2^ = 67%).

Fig. 2b shows the results of the pooled analysis for studies of pre-eclampsia and types 1 and 2 diabetes. There was only one study of type 1 diabetes, reported by Savitz et al in 2014 [22], in which there was no significant association between pre-eclampsia and future incident type 1 diabetes. For incident type 2 diabetes, however, there was a significant increase in risk associated with pre-eclampsia from four pooled studies (RR 2.37 [95% CI 1.37, 4.10], *I* ^2^ = 95%) [22, 24, 27, 34].

### Sensitivity analysis for follow-up time and baseline diabetes and hypertension exclusions

Sensitivity analysis was performed to consider the effect of follow-up time for diabetes and exclusion of baseline diabetes and hypertension (Table 1). There were three studies with less than 1 year of follow-up, two studies with 1–5 years’ follow-up, nine studies with 6–10 years’ follow up and seven studies with more than 10 years’ follow-up (ESM Figs 1–4). For any diabetes, the pooled results showed there was a greater risk of any diabetes in women who had pre-eclampsia compared with those who did not have pre-eclampsia when they were followed up from less than 1 year postpartum, and this effect persisted beyond 10 years of follow-up (<1 year: RR 1.97 [95% CI 1.35, 2.87]; 1–5 years: RR 2.99 [95% CI 2.44, 3.66]; 6–10 years: RR 2.62 [95% CI 1.96, 3.50]; and >10 years: RR 1.95 [95% CI 1.28, 2.97]).

**Table 1:** Sensitivity analysis considering follow-up duration and exclusion of baseline diabetes or hypertension

Sensitivity analysis	PE and risk of outcome	No. studies	RR (95% CI)
Length of follow-up			
<1 year	Adjusted risk of any DM	2	2.17 (0.71, 6.54)
	T1DM	1^a^	1.80 (0.83, 3.92)
	T2DM	1^a^	2.00 (1.25, 3.20)
	Pooled analysis	3	1.97 (1.35, 2.87)
1–5 years	Unadjusted risk of any DM	1	1.32 (0.05, 31.83)
	Adjusted risk of any DM	1	3.00 (2.45, 3.67)
	Pooled analysis	2	2.99 (2.44, 3.66)
6–10 years	Unadjusted risk of any DM	3	7.42 (1.30, 42.30)
	Adjusted risk of any DM	5	2.43 (1.72, 3.44)
	T2DM	1	3.11 (2.10, 4.61)
	Pooled analysis	9	2.62 (1.96, 3.50)
>10 years	Unadjusted risk of any DM	3	1.80 (1.35, 2.42)
	Adjusted risk of any DM	2	1.94 (1.00, 3.77)
	T2DM	2	2.25 (0.90, 5.61)
	Pooled analysis	7	1.95 (1.28, 2.97)
Exclusion of baseline comorbidity			
Exclusion of baseline DM	Unadjusted risk of any DM	5	2.14 (1.62, 2.82)
	Adjusted risk of any DM	7	2.41 (1.88, 3.09)
	T1DM	1^a^	1.80 (0.83, 3.92)
	T2DM	3^a^	2.17 (1.08, 4.37)
	Pooled analysis	15	2.34 (1.86, 2.93)
Exclusion of baseline hypertension	Unadjusted risk of any DM	2	1.97 (0.22, 17.82)
	Adjusted risk of any DM	6	2.55 (1.98, 3.27)
	T1DM	1^a^	1.80 (0.83, 3.92)
	T2DM	1^a^	2.00 (1.27, 3.14)
	Pooled analysis	9	2.40 (1.97, 2.92)

^a^The same study with subgroups of types 1 and 2 diabetes mellitus

DM, diabetes mellitus; PE, pre-eclampsia; T1DM, type 1 diabetes; T2DM, type 2 diabetes

We were able to examine studies for the risk of future type 2 diabetes only. This analysis showed that an increased risk of type 2 diabetes was already apparent after a follow-up of less than 1 year and persisted until 10 years, albeit the number of studies was small. We then concentrated on studies that excluded either baseline diabetes or hypertension from the study cohort. This showed a significantly increased risk in any diabetes in the pre-eclampsia group (diabetes exclusion: aRR 2.34 [95% CI 1.86, 2.93]; hypertension exclusion: aRR 2.40 [95% CI 1.97, 2.92]). The individual Forest plots are shown as supplementary figures (ESM Figs 1–6).

### Sensitivity analysis considering studies that adjusted for BMI, age and gestational diabetes between the pre-eclampsia and control groups

We conducted sensitivity analyses to consider the confounding factors of BMI, age and gestational diabetes (Table 2). We were unable to examine other important confounding factors due to the lack of studies presenting such data. Following adjustment for BMI in the pre-eclampsia and control groups, there was a significantly increased risk of any diabetes in the pooled analysis (aRR 2.38 [95% CI 1.74, 3.24]) and of type 2 diabetes (aRR 2.53 [95% CI 1.64, 3.90]). This increased risk for any diabetes in the pooled analysis remained in studies that also excluded baseline hypertension and diabetes at recruitment (aRR 2.61 [95% CI 1.79, 3.80]). In studies that adjusted for age in the pre-eclampsia and control groups, there was a statistically significant increased risk of both any diabetes in the pooled analysis (aRR 2.35 [95% CI 1.87, 2.95]) and of type 2 diabetes (aRR 2.37 [95% CI 1.37, 4.10]). Further sensitivity analyses were performed with studies that either excluded or adjusted for gestational diabetes. There was a statistically significant increased risk of any diabetes in the pooled analysis (aRR 2.36 [95% CI 1.94, 2.88]). ESM Figs 7–10 show the individual Forest plots for the data presented in Table 2.

**Table 2:** Sensitivity analyses considering the risk of pre-eclampsia and diabetes in studies that adjusted for BMI, BMI with exclusion of baseline hypertension and diabetes, age and gestational diabetes

Adjustment	No. studies	RR (95% CI)
Adjustment for BMI		
Any DM	4	2.41 (1.37, 4.24)
T1DM	1^a^	1.80 (0.83, 3.92)
T2DM	2^a^	2.53 (1.64, 3.90)
Pooled analysis	6	2.38 (1.74, 3.24)
Adjustment for BMI, excluding baseline HTN and DM		
Any DM	2	3.48 (2.37, 5.10)
T1DM	1^a^	1.80 (0.83, 3.92)
T2DM	1^a^	2.00 (1.27, 3.14)
Pooled analysis	3	2.61 (1.79, 3.80)
Adjustment for age		
Any DM, matched	2	6.07 (0.72, 51.38)
Any DM, adjusted	8	2.32 (1.86, 2.91)
T1DM, adjusted	1^a^	1.80 (0.83, 3.92)
T2DM, adjusted	4^a^	2.37 (1.37, 4.10)
Pooled analysis	14	2.35 (1.87, 2.95)
Adjustment for gestational diabetes		
Any DM, excluding GDM, unadjusted	1	2.08 (1.57, 2.75)
Any DM, excluding GDM, adjusted	4	2.96 (2.04, 4.29)
Any DM, adjusted for GDM	1	1.82 (1.26, 2.62)
T1DM, adjusted for GDM	1^a^	1.80 (0.85, 3.80)
T2DM, adjusted for GDM	1^a^	2.00 (1.25, 3.20)
Pooled analysis	7	2.36 (1.94, 2.88)

^a^The same study with subgroups of types 1 and 2 diabetes mellitus

DM, diabetes mellitus; GDM, gestational diabetes; HTN, hypertension; PE, pre-eclampsia; T1DM, type 1 diabetes; T2DM, type 2 diabetes

The full metabolic risk factor profile of the pre-eclampsia and control populations is shown in ESM Table 5. There were significant differences in age, BP and BMI between the pre-eclampsia and control groups at their follow-up in five [11, 12, 21, 26, 30], eight [11, 12, 18, 19, 25, 26, 29, 30] and six [11, 12, 19, 21, 26, 30] out of 21 studies, respectively. However, this population consisted of only 0.5% of the total participating women, as metabolic risk factor profiles were not available in the studies that contributed the majority of participants to this systematic review and meta-analysis [10, 22, 28, 34].

## Discussion

Our systematic review and meta-analysis of 21 studies, including more than 2.8 million women, suggests that there is an association of pre-eclampsia with future incident diabetes. The risk of diabetes in women who had experienced pre-eclampsia was approximately double that of women without a history of pre-eclampsia, and increased to 2.4-fold if type 2 diabetes was considered exclusively. This effect was seen in the first year following delivery and persisted beyond 10 years. Diabetes is a well-known risk factor for pre-eclampsia [36]. However, pre-eclampsia has not been established as a risk factor for future diabetes. In comparison, gestational diabetes is a well-recognised risk factor for future diabetes. Women with pregnancies complicated by gestational diabetes have previously been reported to have a sevenfold increased risk of developing type 2 diabetes compared with those with normoglycaemic pregnancies [37]. Our study therefore extends the literature on the association between pre-eclampsia and diabetes.

Current research supports the link between pre-eclampsia and future diabetes, with several national or regional registry studies with large sample sizes and adjustment for confounding factors all showing similar results [9, 10, 22, 28, 34]. The studies that have not shown an association are mainly those with smaller sample sizes [11, 12, 18, 19, 21, 23, 25, 26, 29, 30]. There are gaps in the current literature, in particular with respect to the link between pre-eclampsia and type 1 diabetes. Furthermore, it is difficult to know whether the association we report here relates to confounding factors. We were unable to fully evaluate the effects of all confounding factors and undertake further sensitivity analyses due to the absence of such data in the studies included in the current meta-analysis. For example, only two studies adjusted for age and BMI, as well as excluding pre-existing diabetes and hypertension in the study populations [22, 32]. Moreover, only seven studies either adjusted for or excluded patients with gestational diabetes [9, 10, 22, 28, 31–33], a known risk factor for future diabetes and pre-eclampsia development [38]. In the few studies where adjustments for age, BMI or gestational diabetes were made (Table 2), the risk of future type 2 diabetes remained increased in women who had pre-eclampsia compared with the control group.

The underlying mechanism for the association between pre-eclampsia and future diabetes is unclear. Pre-eclampsia and diabetes share common risk factors, including age older than 40 years, obesity, hypertension and Afro-Caribbean or South Asian ethnic origin [39, 40]. It may be that women with pre-eclampsia have an underlying predisposition to insulin resistance and the metabolic syndrome, and present with pre-eclampsia as an early indicator of their adverse metabolic phenotype over the life course.

Risk scores allow a non-invasive method of identifying individuals at high risk of future diabetes. The ADA risk tool takes into account age, BMI, hypertension, history of gestational diabetes, family history of diabetes, sex and levels of physical activity [41]. The Finnish Diabetes Risk Score (FINDRISC) [42] is the most commonly used score in Europe, and has been endorsed by the European Society of Cardiology, the EASD [43] and the Public Health Agency of Canada [44]. FINDRISC predicts the 10-year risk of developing type 2 diabetes by considering age, BMI, use of antihypertensive medication, history of hyperglycaemia (including gestational diabetes), family history of diabetes, waist circumference, physical activity, and fruit and vegetable intake [42].

Currently, screening beyond history-taking to identify risk factors for pre-eclampsia during pregnancy is not recommended by the American Congress of Obstetricians and Gynecologists (ACOG). Risk factors recognised by ACOG are: age older than 40 years, obesity, chronic hypertension, diabetes (type 1 or 2), chronic renal disease, previous pre-eclampsia, thrombophilia, systemic lupus erythematosus, primiparity, multiple pregnancy, in vitro fertilisation and a family history of pre-eclampsia [45]. This overlap of risk factors for developing pre-eclampsia and type 2 diabetes could have contributed to the association of pre-eclampsia and future diabetes we report here. Furthermore, there is likely to be interplay between the cardiovascular and metabolic systems. A history of pre-eclampsia is also related to poor future cardiovascular health, while cardiovascular disease is itself a known risk factor for diabetes [46]. In the few studies where adjustments for age or BMI were made (Table 2), the risk of future type 2 diabetes remained increased in women who had pre-eclampsia compared with the control group. Nevertheless, as highlighted above, a number of risk factors are known to significantly increase the risk of future diabetes; none of the studies included in this meta-analysis fully adjusted for all of these risk factors, and so we were unable to undertake further sensitivity analyses.

The strength of our study lies in the number of recent studies included and the large sample size; our meta-analysis of 21 studies examined more than 2.8 million women, including more than 72,500 women with pre-eclampsia with 845,834 patient-years of follow-up. The inclusion of more recent studies means that there is a greater likelihood of the study findings being generalisable to current practice. The majority of the studies were designed to examine future diabetes or insulin resistance and the metabolic syndrome as their main outcome (*n* = 18), with these studies contributing 99% of the women in our meta-analysis.

There are a number of limitations to our analysis. As with any meta-analysis, there is an inherent limitation from publication bias, where studies with positive findings are more likely to be published than those that show neutral outcomes. The majority of women included were from retrospective studies, where there is limited control over the quality of data collected. There may have been inconsistent, incomplete or inaccurate historical data with respect to pre-eclampsia diagnosis, as well as recall bias, which could have caused the incorrect assignment of participants to case and control groups. In addition, five studies used questionnaire data to assess the outcome of diabetes [11, 25, 26, 29, 31]. Finally, it is likely that significant unmeasured confounders may have contributed to our reported association between pre-eclampsia and future diabetes risk, as none of the studies included in this analysis adequately adjusted for all of the risk factors that form the basis of many of the established diabetes risk prediction scores [41, 42].

Given the gaps in the current literature, further work is required to examine the association between pre-eclampsia and type 1 diabetes in particular. There is a need for studies that use propensity-matching methods or more comprehensive adjustments for confounding factors, as well as for high-quality studies with long-term follow-up for outcome events. In addition, mechanistic research is required to further our understanding of the association between pre-eclampsia and future diabetes in order to identify risk-reduction strategies.

Our finding of an association between pre-eclampsia and the future development of incident diabetes is clinically important, as it suggests that formal risk assessment for the future development of diabetes using established risk scores may be considered in pregnant women with pre-eclampsia [41, 42]. Furthermore, clinicians may find it pertinent to enquire about a history of pre-eclampsia as a part of the metabolic and cardiovascular assessment of women or to incorporate into risk-prediction formulas. Since women with pre-eclampsia are already known to be at risk of future cardiovascular disease [47], our study highlights the importance of lifestyle and risk-factor modification, and regular monitoring of BMI and HbA_1c_ in these women to further reduce their cardiovascular and metabolic risks. In line with the ACOG recommendation to perform annual fasting glucose testing following severe pre-eclampsia [45], we recommend a detailed cost–benefit analysis to determine whether and when a screening programme for diabetes in this high-risk population should be initiated.

### Conclusions

Our meta-analysis of 21 studies, which included more than 72,500 women with pre-eclampsia, showed that pre-eclampsia is independently associated with a twofold increase in future diabetes. This increased risk was observed from 1 year following delivery and persisted beyond 10 years postpartum. It is likely that significant unmeasured confounders contribute to the association that we have reported, and that a shared adverse risk factor profile may contribute to both pre-eclampsia and future diabetes risk. As women with pre-eclampsia are already known to be at risk of future cardiovascular disease [47], our study highlights the need for education on risk, advice about lifestyle modifications, and regular monitoring of BMI and HbA_1c_ in women who have had pre-eclampsia. There is also a need to evaluate a screening programme for diabetes in this high-risk population.

## Electronic supplementary material

Below is the link to the electronic supplementary material.

ESM (PDF 8121 kb)

## Abbreviation

ACOG: American Congress of Obstetricians and Gynecologists
FINDRISC: Finnish Diabetes Risk Score

## References

[1] Steegers EA, von Dadelszen P, Duvekot JJ, Pijnenborg R (2010). Pre-eclampsia. Lancet.

[2] Shennan A, Redman C, Cooper C, Milne F (2012). Are most maternal deaths from pre-eclampsia avoidable?. Lancet.

[3] Nelson-Piercy C (2015). Handbook of obstetric medicine.

[4] Kaaja R, Laivuori H, Laakso M, Tikkanen MJ, Ylikorkala O (1999). Evidence of a state of increased insulin resistance in preeclampsia. Metabolism.

[5] Roberts JM (1998). Endothelial dysfunction in preeclampsia. Semin Reprod Endocrinol.

[6] Redman CWG, Sacks GP, Sargent IL (1999). Preeclampsia: an excessive maternal inflammatory response to pregnancy. Am J Obstet Gynecol.

[7] Hotamisligil GS (2006). Inflammation and metabolic disorders. Nature.

[8] Solomon CG, Seely EW (2001). Brief review: hypertension in pregnancy: a manifestation of the insulin resistance syndrome?. Hypertension.

[9] Carr D, Newton K, Utzschneider K (2009). Preeclampsia and risk of developing subsequent diabetes. Hypertens Pregnancy.

[10] Feig DS, Shah BR, Lipscombe LL (2013). Preeclampsia as a risk factor for diabetes: a population-based cohort study. PLoS Med.

[11] Andersgaard AB, Acharya G, Mathiesen EB, Johnsen SH, Straume B, Oian P (2012). Recurrence and long-term maternal health risks of hypertensive disorders of pregnancy: a population-based study. Am J Obstet Gynecol.

[12] Breetveld NM, Ghossein-Doha C, van Kuijk S (2015). Cardiovascular disease risk is only elevated in hypertensive, formerly preeclamptic women. BJOG.

[13] Brown MA, Lindheimer MD, de Swiet M, Van Assche A, Moutquin JM (2001). The classification and diagnosis of the hypertensive disorders of pregnancy: statement from the International Society for the Study of Hypertension in Pregnancy (ISSHP). Hypertens Pregnancy.

[14] World Health Organization (2006). Definition and diagnosis of diabetes mellitus and intermediate hyperglycaemia: report of a WHO/IDF consultation.

[15] Wells G, Shea B, O’Connell D, et al. (2000) The Newcastle-Ottawa Scale (NOS) for assessing the quality of nonrandomised studies in meta-analyses. Available from www.ohri.ca/programs/clinical_epidemiology/oxford.asp

[16] Ioannidis JP, Trikalinos TA (2007). The appropriateness of asymmetry tests for publication bias in meta-analyses: a large survey. CMAJ.

[17] Higgins JPT, Green S (eds) (2008) Cochrane handbook for systematic reviews of interventions. The Cochrane Collaboration. Available from www.cochrane-handbook.org

[18] Drost JT, Arpaci G, Ottervanger JP (2012). Cardiovascular risk factors in women 10 years post early preeclampsia: the Preeclampsia Risk Evaluation in Females Study (PREVFEM). Eur J Prev Cardiol.

[19] Forest J-C, Girouard J, Massé J (2005). Early occurrence of metabolic syndrome after hypertension in pregnancy. Obstet Gynecol.

[20] Magnussen EB, Vatten LJ, Smith GD, Romundstad PR (2009). Hypertensive disorders in pregnancy and subsequently measured cardiovascular risk factors. Obstet Gynecol.

[21] van Rijn BB, Nijdam M-E, Bruinse HW (2013). Cardiovascular disease risk factors in women with a history of early-onset preeclampsia. Obstet Gynecol.

[22] Savitz DA, Danilack VA, Elston B, Lipkind HS (2014). Pregnancy-induced hypertension and diabetes and the risk of cardiovascular disease, stroke, and diabetes hospitalization in the year following delivery. Am J Epidemiol.

[23] Männistö T, Mendola P, Vääräsmäki M (2013). Elevated blood pressure in pregnancy and subsequent chronic disease risk. Circulation.

[24] Libby G, Murphy D, McEwan N (2007). Pre-eclampsia and the later development of type 2 diabetes in mothers and their children: an intergenerational study from the Walker cohort. Diabetologia.

[25] Lazdam M, de la Horra A, Diesch J (2012). Unique blood pressure characteristics in mother and offspring after early onset preeclampsia. Hypertension.

[26] Kaaja R, Kinnunen T, Luoto R (2005). Regional differences in the prevalence of pre-eclampsia in relation to the risk factors for coronary artery disease in women in Finland. Eur Heart J.

[27] Hashemi S, Ramezani Tehrani F, Hasheminia M, Azizi F (2012). Evaluation the risk of metabolic disorder in women with previous preeclampsia participated in Tehran lipid and glucose study. Iran J Endocrinol Metab.

[28] Engeland A, Bjørge T, Daltveit AK (2011). Risk of diabetes after gestational diabetes and preeclampsia. A registry-based study of 230,000 women in Norway. Eur J Epidemiol.

[29] Edlow AG, Srinivas SK, Elovitz MA (2009). Investigating the risk of hypertension shortly after pregnancies complicated by preeclampsia. Am J Obstet Gynecol.

[30] Berends AL, de Groot CJ, Sijbrands EJ (2008). Shared constitutional risks for maternal vascular-related pregnancy complications and future cardiovascular disease. Hypertension.

[31] Callaway LK, Lawlor DA, O’Callaghan M, Williams GM, Najman JM, McIntyre HD (2007). Diabetes mellitus in the 21 years after a pregnancy that was complicated by hypertension: findings from a prospective cohort study. Am J Obstet Gynecol.

[32] Wang I-K, Tsai I-J, Chen P-C (2012). Hypertensive disorders in pregnancy and subsequent diabetes mellitus: a retrospective cohort study. Am J Med.

[33] Tam WH, Ching-wan Ma R, Ozaki R (2015). [189-POS]: cardiometabolic risk among women with a prior history of pre-eclampsia. Pregnancy Hypertens.

[34] Lykke JA, Langhoff-Roos J, Sibai BM, Funai EF, Triche EW, Paidas MJ (2009). Hypertensive pregnancy disorders and subsequent cardiovascular morbidity and type 2 diabetes mellitus in the mother. Hypertension.

[35] Tranquilli AL, Dekker G, Magee L (2014). The classification, diagnosis and management of the hypertensive disorders of pregnancy: a revised statement from the ISSHP. Pregnancy Hypertens.

[36] Garner PR, D’Alton ME, Dudley DK, Huard P, Hardie M (1990). Preeclampsia in diabetic pregnancies. Am J Obstet Gynecol.

[37] Bellamy L, Casas JP, Hingorani AD, Williams D (2009). Type 2 diabetes mellitus after gestational diabetes: a systematic review and meta-analysis. Lancet.

[38] Tamayo T, Tamayo M, Rathmann W, Potthoff P (2016). Prevalence of gestational diabetes and risk of complications before and after initiation of a general systematic two-step screening strategy in Germany (2012-2014). Diabetes Res Clin Pract.

[39] National Institute for Health and Care Excellence (2015). Diabetes in pregnancy. Management of diabetes and its complications from preconception to the postnatal period. NICE Guideline 3.

[40] National Institute for Health and Care Excellence (2010). Hypertension in pregnancy: the management of hypertensive disorders during pregnancy. NICE Clinical Guideline 107.

[41] Bang H, Edwards AM, Bomback AS (2009). Development and validation of a patient self-assessment score for diabetes risk. Ann Intern Med.

[42] Lindstrom J, Tuomilehto J (2003). The diabetes risk score: a practical tool to predict type 2 diabetes risk. Diabetes Care.

[43] Ryden L, Grant PJ, Anker SD (2014). ESC guidelines on diabetes, pre-diabetes, and cardiovascular diseases developed in collaboration with the EASD—summary. Diab Vasc Dis Res.

[44] Canadian Task Force on Preventive Health Care (2012). Recommendations on screening for type 2 diabetes in adults. CMAJ.

[45] ACOG (2013). Hypertension in pregnancy. Report of the American College of Obstetricians and Gynecologists’ Task Force on Hypertension in Pregnancy. Obstet Gynecol.

[46] Gress TW, Nieto FJ, Shahar E, Wofford MR, Brancati FL (2000). Hypertension and antihypertensive therapy as risk factors for type 2 diabetes mellitus. Atherosclerosis Risk in Communities Study. N Engl J Med.

[47] Bellamy L, Casas J-P, Hingorani AD, Williams DJ (2007). Pre-eclampsia and risk of cardiovascular disease and cancer in later life: systematic review and meta-analysis. BMJ.

