# Supplementary material for: Pre-eclampsia is associated with a twofold increase in diabetes: a systematic review and meta-analysis
Source: Diabetologia. 2016 Sep 19;59(12):2518–26. doi: 10.1007/s00125-016-4098-x (PMC6518071; doi:10.1007/s00125-016-4098-x)
Supplement: Supplementary file 1 — (PDF 8121 kb) [file 125_2016_4098_MOESM1_ESM.pdf]

ESM Methods. Search Terms.

Synonyms of pre-eclampsia (‘preeclampsia’ or ‘pre-eclampsia’ or ‘EPH’ or ‘pregnancy toxemia’ or ‘edema-proteinuria-hypertension gestos’) AND ‘hypertension’ or ‘diabetes’ or ‘ischaemic heart disease’ or ‘ischemic heart disease’ or ‘coronary artery disease’ or ‘coronary heart disease’ or ‘myocardial infarction’ or ‘acute coronary syndrome’ or ‘heart failure’ or ‘cardiac failure’ or ‘left ventricular systolic dysfunction’ or ‘stroke’ or ‘cerebrovascular disease’ or ‘cerebrovascular accident’ or ‘pulmonary embolus’ or ‘venous thromboembolism’ or ‘deep vein thrombosis’ or ‘cardiomyopathy’ or ‘renal impairment’ or ‘kidney disease’ or ‘peripheral vascular disease’.

To ensure a comprehensive search strategy, we also searched for synonyms of hypertensive disorders of pregnancy (‘pregnancy induced hypertension’ or ‘pregnancy-induced hypertension’ or ‘hypertensive disorder$ pregnancy’ or ‘hypertensive disorder$ of pregnancy’ or ‘hypertensive disorder$ in pregnancy’ or ‘hypertensive disorder$ complicating pregnancy’ or ‘hypertension in pregnancy’ or ‘hypertension pregnant women’ or ‘hypertension pregnancy’ or ‘hypertension pregnancy-induced’ or ‘pregnancy hypertension’ or ‘hypertensive pregnancy disorder$’ or ‘pregnancy-related hypertensive disorder$’) AND diabetes.

ESM Table 1. Study design and participant characteristics.

**Study ID**	**Study Design; Country; Year**	**Total No. of Women (PE/no PE)**	**Mean Age**	**Parity**	**Participant Selection Criteria**	**Outcomes Assessed**
Andersgaard et al (2012) [11]	Cross-sectional study; Norway; 1994-1995.	8,088 (PE 901, control 7,187)	23.4[a]	A	Women in the Tromso study which focuses on cardiovascular risk factors.	Any diabetes or use of antidiabetic medication, self-reporting on questionnaire.
Callaway et al (2007) [31]	Prospective cohort study; Australia, 1981-1984	3,639 (PE 333, control 3,306)	25[a]	A	Women in the Mater-University of Queensland Study of Pregnancy between 1981-1984.	Any diabetes, self-reporting on questionnaire.
Libby et al (2007) [24]	Prospective cohort study; Scotland; 1952-2003.	7,187 (PE 810, control 6,377)	Median of 25 and 26[a]	A	Women in the Walker Database, which included the majority of women delivering in Dundee between 1952-1966.	T2DM, confirmed by manual validation of case records.
Kaaja et al (2005) [26]	Retrospective cross-sectional study; Finland; 2002.	3559 (PE 397, control 3,162)	26.7[a]	A	Women in FINRISK-cross sectional survey which monitors cardiovascular risk factors in Finland every 5 years.	Any diabetes, self-reporting on questionnaire.
Mannisto et al (2013) [23]	Prospective cohort study; Finland; 1966-2006.	6,794 (PE 242, control 6,552)	26.7[a]	A	Women in the prospective Northern Finland Birth Cohort 1966, which composed of all expected births in 1966.	Any diabetes, ascertained by ICD codes.
Lykke et al (2009) [34]	Retrospective cohort study; Denmark; 1978-2007.	774,838 (PE 33,826, control 741,012)	26.8[a]	P	Women age 15-50 who had first delivery from 1978-2007 in the National Patient Registry in Denmark.	T2DM, obtained from the National Patient Registry in Denmark.

Forest et al (2005) [19]	Prospective case-control study; Canada; 1989-1997.	231 (PE 63, control 168)	27.2[a]	P	Women in previous prospective studies for biochemical and sonographic markers of PE and matched controls.	Fasting blood glucose ≥7mmol/l, blood sampled in research clinic.
Edlow et al (2009) [29]	Prospective case-control study; USA; 2005-2007.	219 (PE 79, control 140)	27.5[a]	A	Women in the Pre-eclampsia: Mechanisms and Consequences study from Hospital of the University of Pennsylvania between March 2005-August 2007.	Any diabetes, assessed through telephone questionnaire.
Berends et al (2008) [30]	Case-control study; Netherlands; 1983-2004.	153 (PE 47, control 106)	27.7[a]	A	Women with a history of PE recruited from the Genetic Research in Isolated Populations Study.	Any diabetes, participants examined at research centre.
Wang et al (2012) [32]	Retrospective cohort study; Taiwan; 1997-2008	5,178 (PE 651, control 4,527).	29[a]	A	Random subset from National Health Insurance Research Database 1997-2003.	Any diabetes, ascertained by ICD-9.
Drost et al (2012) [18]	Retrospective cohort study; Netherlands; 1991-2007.	671 (PE 339, control 332)	29.2[a]	A	Women delivered at the Isala Klinieken in Zwolle, The Netherlands between 1991-2007 with and without PE.	Any diabetes, ascertained by trained nurses at cardiology clinic.
Van Rijn et al (2013) [21]	Prospective cross-sectional study; Netherlands; 1994-2007.	617 (PE 243, control 374)	29.4[a]	P	Women with a first pregnancy complicated by early onset PE in a tertiary centre in the Netherlands, versus the control group from a study that comprises an unselected population-based cohort of similar age, demographics, and geographical background.	Any diabetes, assessed in research clinic.
Feig et al (2013) [10]	Retrospective cohort study; Canada; 1994-2008.	948,035 (PE 22,933, control 925,102)	29.5[a]	A	Linkage of administrative health claims for public health insurance with the Canadian Institute for Health	Any diabetes, through health insurance claims.

					Information Discharge Abstract Database for delivery information.	
Carr et al (2009) [9]	Retrospective cohort study; USA; 1985-2002.	31,463 (PE 2,032, control 29,431)	30.1[a]	A	Women with and without PE in Group Health, a Washington state health plan, linked to subsequent automated data for the diagnosis of diabetes (using ICD-9 codes).	Any diabetes, via ICD-9 codes, laboratory and pharmacy records.
Lazdam et al (2012) [25]	Prospective cohort study; England; 1998-2003.	140 (PE 90, control 50)	30.4[a]	A	Women who were discharged from Oxford Maternity Unit between 1998-2003.	Any diabetes, self-reporting on questionnaire.
Engeland et al (2011) [28]	Prospective cohort study; Norway; 2004-2008.	226,832 (PE 8,822, control 218,010)	31[a]	A	Women with pregnancies registered in the Medical Birth Registry of Norway during 2004–2008.	Use of antidiabetic medication, using national prescription data from pharmacies.
Breetveld et al (2014) [12]	Retrospective cohort study; Netherlands; 2010-2012.	165 (PE 115; control 50)	37.5[b]	A	Recruitment from a database of women who had PE and volunteered to participate in a cardiovascular follow-up study program.	Any diabetes, determined by researcher.
Magnussen et al (2009) [20]	Prospective cohort study; Norway; 1967-1995.	15,065 (PE 661, control 14,404)	40[b]	P	Women in the Nord-Trondelag Health (HUNT) study who had first singleton pregnancies from 1976-1995.	Any diabetes, self-reporting on questionnaire then validated by fasting blood glucose.
Hashemi et al (2012) [27]	Prospective cohort study; Iran; unclear.	452 (PE 226, control 226)	Unclear	A	Women in the Tehran Lipid Glucose Study which is on disease risk factors.	T2DM, confirmed by oral glucose tolerance test.
Savitz et al (2014) [22]	Retrospective cohort study; USA; 1995-2004.	849,639 (no data on numbers in the PE cohort)	Unclear	A	Data on all births in hospitals in New York City obtained by linking birth certificates to hospital discharge data.	T1DM and T2DM, ascertained by ICD-9 codes.

Tam et al (2015) [33]	Case-control study; Hong Kong; unclear.	693 (PE 50, controls 643)	Unclear	A	Women in the Hyperglycemia and Adverse Pregnancy Outcome study, a multinational study.	Any diabetes, assessed by oral glucose tolerance test.

A=any parity, HTN=hypertension, P=primiparous, PE=pre-eclampsia. T1DM=type 1 diabetes, T2DM=type 2 diabetes. [a]At index pregnancy. [b]At follow-up.

ESM Table 2. Study quality assessment overview.

Study ID	Selection				Comparability	Outcome			Total Score
	Representative of the exposed cohort	Selection of the non-exposed cohort	Ascertainment of exposure	Demonstration that outcome of interest was not present at start of study	Comparability of cohort	Assessment of outcome	Follow-up duration to capture outcomes	Adequacy of follow-up	
Andersgaard et al (2012) [11]	*	*					*	*	4
Callaway et al (2007) [31]	*	*	*	*			*		5
Libby et al (2007) [24]	*	*	*	*	*	*	*		7
Kaaja et al (2005) [26]	*	*	*			*	*	*	6
Mannisto et al (2013) [23]	*	*	*	*	**	*	*		8
Lykke et al (2009) [34]	*	*	*	*	*	*	*	*	8
Forest et al (2005) [19]	*	*	*	*	*	*		*	7
Edlow et al (2009) [29]	*	*	*		*				4
Berends et al (2008) [30]	*	*	*	*		*		*	6
Wang et al (2012) [32]	*	*	*	*	**	*		*	8
Drost et al (2012) [18]	*		*		*	*	*		5

Van Rijn et al (2013) [21]	*		*		*	*			4
Feig et al (2013) [10]	*	*	*	*	*	*		*	7
Carr et al (2009) [9]	*	*	*	*	**	*		*	8
Lazdam et al (2012) [25]	*		*	*	*	*	*		6
Engeland et al (2011) [28]	*	*	*	*	*	*		*	7
Breetveld et al (2014) [12]	*	*	*	*		*			5
Magnussen et al (2009) [20]	*	*	*	*	*	*	*		7
Hashemi et al (2012) [27]	*				*	*	*		4
Savitz et al (2014) [22]	*	*	*	*	**	*		*	8
Tam et al (2015) [33]	*	*	*	*	*	*	*		7

ESM Table 3. Study quality assessment in detail.

Study ID	Representative of the exposed cohort	Selection of the non-exposed cohort	Ascertainment of exposure	Demonstration that outcome of interest was not present at start of study	Comparability of cohort	Assessment of outcome	Follow-up duration to capture outcomes	Adequacy of follow-up
Andersgaard et al (2012) [11]	General cohort of women.	Controls from same cohort.	Completed questionnaires.	No exclusions.	Unadjusted	Self-reported diabetes or use of antidiabetic medication.	Mean 24.7 years.	434/10,408 (4%) loss to follow-up.
Callaway et al (2007) [31]	General cohort of women.	Controls from the same cohort.	Identified from previous study.	Excluded diabetes and gestational diabetes.	Unadjusted.	Self-reported diabetes.	21 years.	3,639/7,173 (51%) did not complete questionnaire.
Libby et al (2007) [24]	General cohort of women.	Controls from same cohort.	From database.	Excluded T1DM.	Adjusted for age, socioeconomic status, birthweight of the offspring.	From use of medicines database (1980-1993) and the diabetic database on all diabetics in the area (from 1993).	Median 46 years.	1,192/8,384 (14%) had died or moved from study area.
Kaaja et al (2005) [26]	General cohort of women.	Controls from same cohort.	Completed questionnaires with trained staff.	No exclusions.	Unadjusted	Completed questionnaire with trained staff.	Mean 17.4 years.	>90% came for the assessments with trained staff at their local health care centre.
Mannisto et al (2013) [23]	General cohort of women.	Controls from same cohort.	Medical records reviewed by 2 obstetricians.	Excluded diabetes.	Adjusted for BMI, smoking parity and socioeconomic status.	ICD codes recorded in Finnish registers.	Mean 39.4 years.	1,565/12,055 (13%) had missing blood pressures or died.

Lykke et al (2009) [34]	General cohort of women.	Controls from same cohort.	Data from national database.	Excluded diabetes.	Adjusted for age, year of delivery, preterm delivery, placental abruption, small-for-gestational-age offspring and stillbirth.	From the National Patient Registry in Denmark.	Median 14.6 years.	24,778/807,065 (3%) died or emigrated.
Forest et al (2005) [19]	General cohort of primiparous women.	Matched controls for maternal age and year of index delivery from same cohort.	PE assessed by 1 senior obstetrician.	Excluded diabetes.	Unadjusted, but matched for age.	Assessed at research clinics run by research nurses.	Mean 7.8 years.	No loss to follow-up.
Edlow et al (2009) [29]	General cohort of women with diagnosis of PE.	General cohort of women without diagnosis of PE.	Women identified from previous study.	No exclusions.	Adjusted for ethnicity, BMI, parity.	Assessed through a telephone questionnaire.	6-13 months after delivery.	Out of eligible patients, participated by PE 79 /113 (70%) and control 140/239 (59%) women.
Berends et al (2008) [30]	General cohort of women from the Genetic Research in Isolated Populations study, where all participants were of White origin.	Controls from the Erasmus Rucpphen Family study, a substudy of the Genetic Research in Isolated Populations study	A research physician reviewed the medical charts.	Excluded diabetes.	Unadjusted.	All participants were examined at research centre.	Median 7.1 years.	Participated by 153/156 (98%), exclusion due to pregnancy.

Wang et al (2012) [32]	General cohort of women.	Matched by age and year of pregnancy from the same cohort.	From database.	Excluded diabetes and gestational diabetes.	Adjusted for age, occupation, obesity and hyperlipidemia.	Identified using ICD-9 codes.	Mean 8.2 years.	Database study.
Drost et al (2012) [18]	General cohort of women.	Age matched controls.	Database to identify women with PE.	No exclusions.	Adjusted for age, years post-index pregnancy and current smoking.	Ascertained by trained nurses.	Mean 10.0 years.	Out of eligible participants, participated by PE 339/448 (76%) and control 332/617 (54%) women.
Van Rijn et al (2013) [21]	General cohort of primiparous women.	Similar age controls.	Women identified from previous studies.	No exclusions.	Adjusted for age and oral contraceptive use.	Assessed at research clinic.	Mean 9.4 months.	Unclear.
Feig et al (2013) [10]	General cohort of women.	Controls from same cohort.	Data from national database.	Excluded diabetes and gestational diabetes.	Adjusted for age, socioeconomic status, hypertension prior to pregnancy, and comorbidity.	Identified through health insurance claims.	Median 8.5 years.	Database study.
Carr et al (2009) [9]	General cohort of women.	Controls from same cohort.	From discharge codes.	Excluded diabetes.	Adjusted for age, primigravidity and gestational diabetes.	Used ICD-9 codes, laboratory and pharmacy records.	Median 8.2 years.	Database study.
Lazdam et al (2012) [25]	General cohort of women.	Match for age, parity, and year of delivery..	Extracted from medical records.	Excluded diabetes.	Unadjusted but matched for age and parity.	Completed questionnaire with research midwife.	9.75 years.	Out of eligible participants, 140/618 (23%) participated.
Engeland et al (2011) [28]	General cohort of women.	Controls from same cohort.	Data from national database.	Excluded diabetes and gestational diabetes.	Adjusted for age and parity.	Use of national prescription data from pharmacies to	Mean 3.7 years.	Database study.

						identify those newly started on antidiabetic medication. Medication dispensed at hospitals were not included.		
Breetveld et al (2014) [12]	General cohort of women.	Women in control group had to be between 25 and 45 years old and to have had their first pregnancy 5-10 years earlier.	Women identified from previous study.	Excluded diabetes.	Unadjusted.	Assessed at research facility.	Mean 5.4 years (PE) and 8.0 years (control).	Unclear.
Magnussen et al (2009) [20]	General cohort of women.	Controls from same cohort.	Data from national database.	Excluded diabetes.	Adjusted for age, duration between index delivery and HUNT study, education, smoking, BMI, and whether receiving social security benefit.	Fasting blood glucose taken to confirm diabetes.	Mean 16.5 years.	Unclear.
Hashemi et al (2012) [27]	General cohort of women.	Age and BMI matched controls.	Completed questionnaires.	No exclusions.	Unadjusted but matched for age and BMI.	Oral glucose tolerance test.	10 years.	Unclear.
Savitz et al (2014) [22]	General cohort of women.	Controls from same cohort.	Use of hospital discharge information.	Excluded diabetes.	Adjusted for year, age, ethnicity, health	Identified using ICD-9 codes.	Within 1 year.	Database study.

					insurance, gestational diabetes, parity, socioeconomic status, smoking, prenatal care and pre-pregnancy weight.			
Tam et al (2015) [33]	General cohort of women	Controls from same cohort.	Identified from previous study.	Excluded diabetes and gestational diabetes.	Adjusted for unclear variables.	Oral glucose tolerance test.	7-11 years.	Unclear.

BMI=body mass index, PE=pre-eclampsia, T1DM=type 1 diabetes.

ESM Table 4. Method of determining pre-eclampsia, outcomes and results.

Study ID	Definition of PE	Timing of outcome assessment	Results
Andersgaard et al (2012) [11]	Self-reported gestational hypertension and proteinuria.	Mean 24.7 years follow-up.	PE vs control: DM 17/901 vs 107/7,187 .
Callaway et al (2007) [31]	Diastolic BP >90 mmHg on 2 occasions associated with proteinuria or excessive fluid retention after 20 weeks gestation.	21 years follow-up.	PE vs control: DM: 51/333 vs 244/3,306.
Libby et al (2007) [24]	Diastolic BP ≥90 mmHg on ≥2 occasions separated by 1 day and albuminuria.	Median 46 years follow-up.	PE vs control: T2DM: 107/810 vs 703/6,377, aOR 1.40 (1.12-1.75).
Kaaja et al (2005) [26]	ISSHP (2014) definition.	Mean 17.4 years follow-up.	PE vs control: DM: 13/397 vs 54/3,162
Mannisto et al (2013) [23]	≥145/95 mmHg with proteinuria ≥0.3 g/l after 20 weeks gestation..	Mean 39.4 years follow-up.	PE vs control: DM: 22/242 vs 388/6,552, HR 1.42 (0.92-2.19).
Lykke et al (2009) [34]	ISSHP (2014) definition.	Median 14.6 years follow-up.	PE vs control: T2DM: Mild PE: 742/26,810 vs 5,604/741,012, aHR 3.53 (3.23-3.85). Severe PE: 177/7,016 vs 5,604/741,012, aHR 3.68 (3.04-4.46).
Forest et al (2005) [19]	ISSHP (2014) definition.	Mean 7.8 years follow-up.	PE vs control: DM: 2/63 vs 0/168.
Edlow et al (2009) [29]	BP ≥140/90 mmHg on 2 occasions ≥6 hours apart or BP ≥160/105, with or without proteinuria.	6-13 months after delivery.	PE vs control: DM: 6/79 vs 5/140, aOR 1.84 (0.5-6.5).
Berends et al (2008) [30]	ISSHP (2014) definition.	Median 7.1 years follow-up.	PE vs control : DM: 2/47 vs 0/106.
Wang et al (2012) [32]	PE defined by ICD-9 codes.	Mean 8.2 years follow-up.	PE vs control: DM: 31/651 vs 31/4,527, aHR 4.15 (2.48-6.95).
Drost et al (2012) [18]	ISSHP (2014) definition.	Mean 10.0 years follow-up.	PE (n=339) vs control (n=332): DM: aOR 1.72 (0.54-5.48).
Van Rijn et al (2013) [21]	ISSHP (2014) definition and required delivery <34 weeks gestation.	Mean 9.4 months follow-up.	PE vs control: DM: 3/243 vs 2/374, aOR 3.67 (0.38-35.64).

Feig et al (2013) [10]	From hospitalization records and outpatient data from physicians' services claims.	Median 8.5 years follow-up.	PE vs control: DM: 1,510/22,933 vs 23,108/925,102, aHR 2.08 (1.97-2.19).
Carr et al (2009) [9]	PE defined by ICD-9 codes.	Median 8.2 years follow-up.	PE (n=2,032) vs control (n=29,431): DM: aHR 1.82 (1.26-2.62).
Lazdam et al (2012) [25]	ISSHP (2014) definition.	Mean 9.75 years follow-up.	PE vs control: DM: 2/90 vs 0/50.
Engeland et al (2011) [28]	ISSHP (2014) definition.	Mean 3.7 years follow-up.	PE (n=8,822) vs control (n=218,010): Drugs to treat DM: aRR 3.0 (2.4-3.6). Both insulin and oral antidiabetics: aRR 4.2 (1.6-11). Oral antidiabetics: aRR 3.0 (2.4-3.7). Insulin only: aRR 2.5 (1.4-4.5).
Breetveld et al (2014) [12]	ISSHP (2014) definition.	Mean 5.4 years (PE) and 8.0 years (control) follow-up.	PE vs control: DM 1/115 vs 0/50.
Magnussen et al (2009) [20]	ISSHP (2014) definition.	Mean 16.3 years (PE) and 16.6 years (control) follow-up.	PE (n=661) vs control (n=14,404): DM: aOR 2.8 (1.6-5.0).
Hashemi et al (2012) [27]	ISSHP (2014) definition.	10 years follow-up.	PE vs control: T2DM: 84/226 vs 27/226.
Savitz et al (2014) [22]	PE defined by ICD-9 codes.	Within 1 year follow-up.	PE vs control: T1DM (n=71): aOR 1.8 (0.8-3.8). T2 DM (n=212): aOR 2.0 (1.3-3.2).
Tam et al (2015) [33]	PE not defined.	7-11 years follow-up.	PE vs controls: DM: 6/50 vs 3/643, aOR 13.0 (1.9-81.0).

DM=diabetes mellitus, ISSHP=International Society for the Study of Hypertension in Pregnancy, HTN=hypertension, PE=pre-eclampsia, T1DM=type 1 diabetes, T2DM=type 2 diabetes.

ESM Table 5. Metabolic risk factor profile of PE and control groups in the included studies. [a]Total PE vs. control.

Study ID	Risk factor profile	During pregnancy			At follow-up		
		PE	Control	*p* value	PE	Control	*p* value
Andersgaard 2012	Age (year)	-	-	-	48.8	47.4	<0.01
	MAP (mmHg)	-	-	-	100	94	<0.01
	BMI (kg/m^2)	-	-	-	26	25	<0.001
	Waist circumference (cm)	-	-	-	87	84	<0.001
	Total cholesterol (mmol/l)	-	-	-	6.12	6.04	<0.05
	HDL (mmol/l)	-	-	-	1.61	1.65	<0.01
	Triacylglycerol (mmol/l)	-	-	-	1.43	1.46	<0.001
	HTN >140/90 (%)	-	-	-	25	13	<0.001
	Angina/MI/stroke (%)	-	-	-	7.7	4.2	<0.001
	BMI>30 (%)	-	-	-	17	10	N.S
	Smoking (%)	-	-	-	32	38	N.S
	FH first degree CVD (%)	-	-	-	64.9	54.8	N.S
	FH first degree DM (%)	-	-	-	19.2	16.2	N.S
Callaway 2007	Not available	-	-	-	-	-	-
Libby 2007	Age (year)	25	26	N.S	71	71	N.S
Kaaja 2005	Age (year)	-	-	-	47.9	46.4	0.006
	HTN in last 12 months	-	-	-	31.8	12.4	<0.001
	HTN ever	-	-	-	73.8	32.7	<0.001
	Antihypertensives, ever used (%)	-	-	-	52.9	29.2	<0.001
	BMI (Kg/m^2)	-	-	-	27.7	26.2	<0.001

	Alcohol (g/previous week)	-	-	-	30.8	37.5	0.027
	Increased cholesterol, ever (%)	-	-	-	39.0	31.4	0.006
	Angina in last 12 months	-	-	-	2.5	0.8	<0.001
	Cardiac insufficiency in last 12 months (%)	-	-	-	2.9	0.7	<0.001
	Smoking (%)	-	-	-	21.5	22.5	N.S
	Cancer (%)	-	-	-	0.8	0.7	N.S
	Cholesterol (mmol/l)	-	-	-	5.4	5.4	N.S
	Use of lipid-lowering medication (%)	-	-	-	3.5	2.4	N.S
Mannisto 2013	BMI (Kg/m^2)	23.5	22.6	<0.0001	-	-	-
	Primiparous (%)	55.0	30.9	<0.0001	-	-	-
	Smoking (%)	18.2	23.8	<0.05	-	-	-
	Age (%)	26.7	26.6	N.S	-	-	-
	Socioeconomic status, managerial (%)	15.3	13.4	N.S	-	-	-
Lykke 2009	Not available	-	-	-	-	-	-
Forest 2005	Age (year)	27.4	27.0	N.S	35.5	35.1	N.S
	BMI (Kg/m^2)	23.3	21.9	0.008	26.9	24.7	0.002
	SBP (mmHg)	-	-	-	114.8	107.9	<0.001
	DBP (mmHg)	-	-	-	75	70	<0.001
	Waist circumference (cm)	-	-	-	82.5	76.9	<0.001
	Waist/Hip ratio	-	-	-	0.79	0.77	0.03
	LDL (mmol/l)	-	-	-	2.90	2.65	0.05
	Apolipoprotein B (g/l)	-	-	-	0.87	0.79	0.02
	Atherogenic index	-	-	-	3.8	3.4	0.03
	FH HTN (%)	-	-	-	65	32	<0.001
	Total cholesterol (mmol/l)	-	-	-	4.77	4.54	N.S
	HDL (mmol/l)	-	-	-	1.33	1.42	N.S

	Triacylglycerol (mmol/l)	-	-	-	1.18	1.02	N.S
	Smoking (%)	-	-	-	24	31	N.S
	Alcohol (%)	-	-	-	3	5	N.S
	Exercise (%)	-	-	-	17	33	N.S
	Oral contraceptive use (%)	-	-	-	18	20	N.S
	FH of CVD <55 years old (%)	-	-	-	27	17	N.S
	FH of DM (%)	-	-	-	18	21	N.S
Edlow 2009	Chronic hypertension (%)	15.2	5.7	0.01	-	-	-
	African American (%)	77	60.7	0.02	-	-	-
	History of PE >1 pregnancy (%)	20.3	5.7	0.02	-	-	-
	Mean age (year)	26.6	28.3	N.S	-	-	--
	Mean BMI (%)	29.3	29.3	N.S	-	-	-
	Smoking (%)	8.8	14.3	N.S	-	-	-
	Primiparous (%)	54	42	N.S	-	-	-
	HTN/antihypertensive use excluding chronic hypertensives (%)	-	-	-	38.7	4.4	<0.001
	BMI >30 (%)	-	-	-	48.7	29.3	N.S
	Dyslipidaemia/lipid-lowering medicine use (%)	-	-	-	8.0	3.1	N.S
Berends 2008	Age (year)	29.2	26.2	<0.001	36.2	39.2	<0.01
	Antihypertensives (%)				19.1	0.9	<0.001
	Median BMI (kg/m^2)	-	-	-	27.2	24.2	<0.01
	Low educational level (%)	-	-	-	38.0	72.6	<0.001
	Smoking (%)	-	-	-	22.0	49.1	<0.001
	Lipid-lowering drugs (%)	-	-	-	2.1	0.9	N.S

	Alcohol consumption (%)	-	-	-	32.0	31.1	N.S
Wang 2012	Not available	-	-	-	-	-	-
Drost 2012	Age (year)	29.8	28.6	<0.05	-	-	-
	Primiparous (%)	79.6	70.2	<0.05	-	-	-
	Smoking (%)	11.2	16.6	N.S	-	-	-
	HTN (%)	-	-	-	43.1	17.2	<0.05
	FH of cardiovascular risk (%)	-	-	-	75.5	63.9	<0.05
	Antihypertensives (%)	-	-	-	20.6	2.1	<0.05
	Current smoking (%)	-	-	-	15.6	17.5	N.S
	Previous smoking (%)	-	-	-	29.5	30.4	N.S
	Adequate control of BP on medication (%)	-	-	-	38.6	14.3	N.S
	Hypercholesterolaemia (%)	-	-	-	38.6	42.5	N.S
	Statin use (%)	-	-	-	1.2	0.3	N.S
Van Rijn 2013	Age (year)	-	-	-	30.5	28.3	<0.001
	SBP (mmHg)	-	-	-	126	120	<0.001
	DBP (mmHg)	-	-	-	79	70	<0.001
	BMI (Kg/m^2)	-	-	-	26.1	24.3	<0.001
	Total cholesterol (mg/dl)	-	-	-	198	186	<0.001
	HDL cholesterol (mg/dl)	-	-	-	55	61	<0.001
	LDL cholesterol (mg/dl)	-	-	-	119	104	<0.001
	Triacylglycerol (mg/dl)	-	-	-	121	108	0.009
	Ratio of total cholesterol to HDL cholesterol	-	-	-	3.81	3.21	<0.001
	Current oral contraceptive use (%)	-	-	-	82	34	<0.001

	White race (%)	-	-	-	99	98	N.S
	Smoking (%)	-	-	-	25.1	27.2	N.S
Feig 2013	Age (year)	29.51	29.54	N/A	-	-	-
	Prior HTN (%)	6.4	1.3	N/A	-	-	-
	Chronic medical unstable comorbidity (%)	12.4	10.2	N/A	-	-	-
	Chronic medical stable comorbidity (%)	27.1	22.2	N/A	-	-	-
	Income quartile 1 (lowest) (%)	21.4	21.8	N/A	-	-	-
Carr 2009	Gestational DM (%)	5.7	4.2	N/A	-	-	-
	Mean age at delivery (year)	30.0	30.1	N/A	-	-	-
Lazdam 2012	Age (year)	E 39.78	30.12	N.S	E 39.78	40.51	N.S
		L 30.04	-	N.S	L 40.04	-	-
	Primiparous (%)	E 80	80	N.S	-	-	-
		L 82	-	N.S	-	-	-
	HTN (%)	-	-	-	E 6	0	0.03[a]
		-	-	-	L 2		
	LDL (mmol/l)	-	-	-	E 2.89	2.61	0.04[a]
		-	-	-	L 2.96	-	-
	Total:HDL cholesterol ratio	-	-	-	E 3.53	2.95	0.002[a]
		-	-	--	L 3.30	-	-
	Triacylglycerol (mmol/l)	-	-	-	E 1.19	0.9	0.05[a]
		-	-	-	L 1.02	-	-

	HOMA-IR	-	-	-	E 2.08	1.52	0.01[a]
		-	-	-	L 2.01	-	-
	Smoking (%)	-	-	-	E 12.5	4.2	N.S
					L 2.3	-	-
Engeland 2011	Not available	-	-	-	-	-	-
Breetveld 2014	Age (year)	-	-	-	36	39	<0.001
	SBP (mmHg)	-	-	-	117	110	<0.01
	MAP (mmHg)	-	-	-	86	82	<0.01
	BMI >30 (%)	-	-	-	18	4	<0.05
	Alcohol (%)	-	-	-	23	72	<0.01
	Smoking	-	-	-	8	10	N.S
	FH of CVD	-	-	-	43	44	N.S
	DBP (mmHg)	-	-	-	10	7	N.S
Magnussen 2009	Age (year)	-	-	-	40.1	39.9	N/A
	Current antihypertensive	-	-	-	9.6	2.2	N/A
	Smoking	-	-	-	26.4	37.3	N/A
	Education 14+ years (%)	-	-	-	10.1	9.4	N/A
Hashemi 2012	Not available	-	-	-	-	-	-
Savitz 2014	Not available	-	-	-	-	-	-
Tam 2015	Not available	-	-	-	-	-	-

BMI=body mass index, CVD=cardiovascular disease, DBP=diastolic blood pressure, DM=diabetes mellitus, E=PE in early pregnancy, FH=family history, HDL=high density lipoprotein, HTN=hypertension, HOMA-IR=homeostatic model assessment-insulin resistance, L=PE in late pregnancy, LDL=low density lipoprotein, MAP=mean arterial pressure, MI=myocardial infarction, N.S=non-significant, PE=pre-eclampsia, SBP=systolic blood pressure.

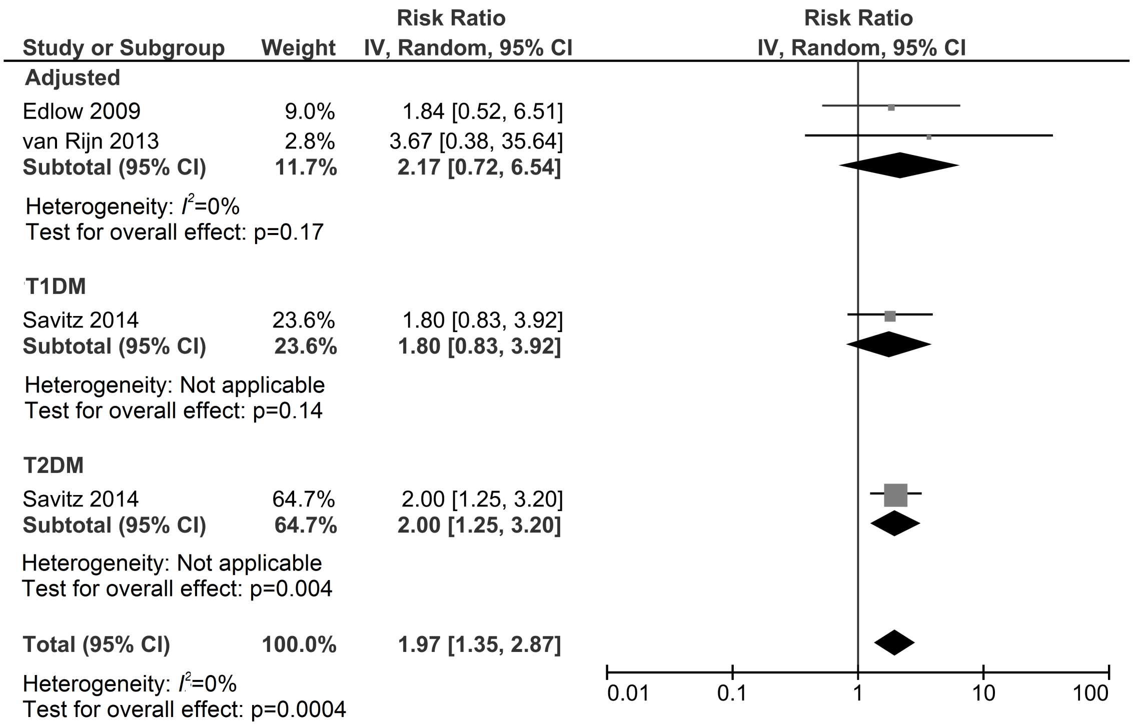

ESM Figure 1. Sensitivity analysis of studies with follow-up <1 year.

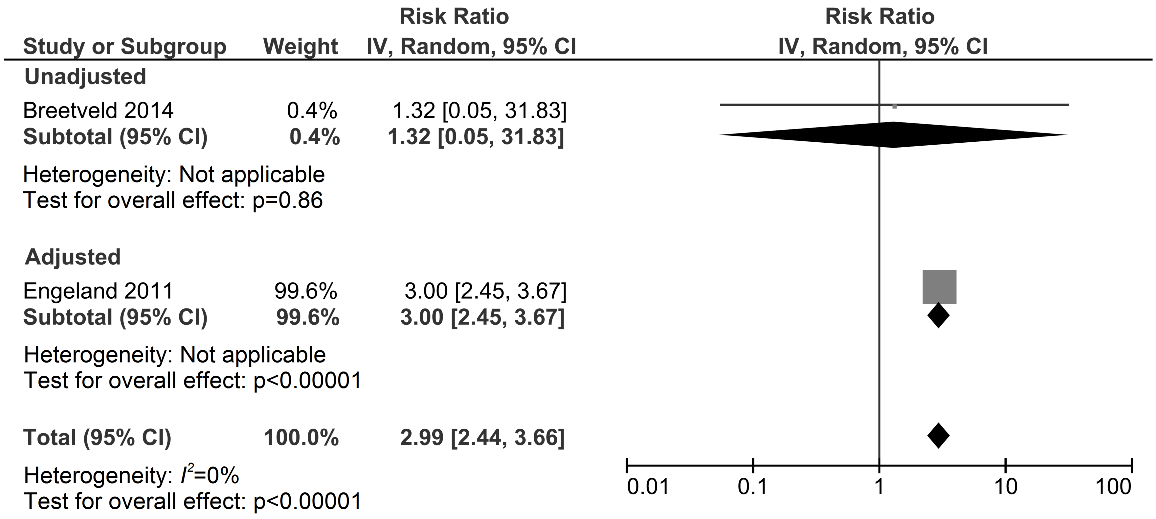

ESM Figure 2. Sensitivity analysis of studies with follow-up 1-5 years.

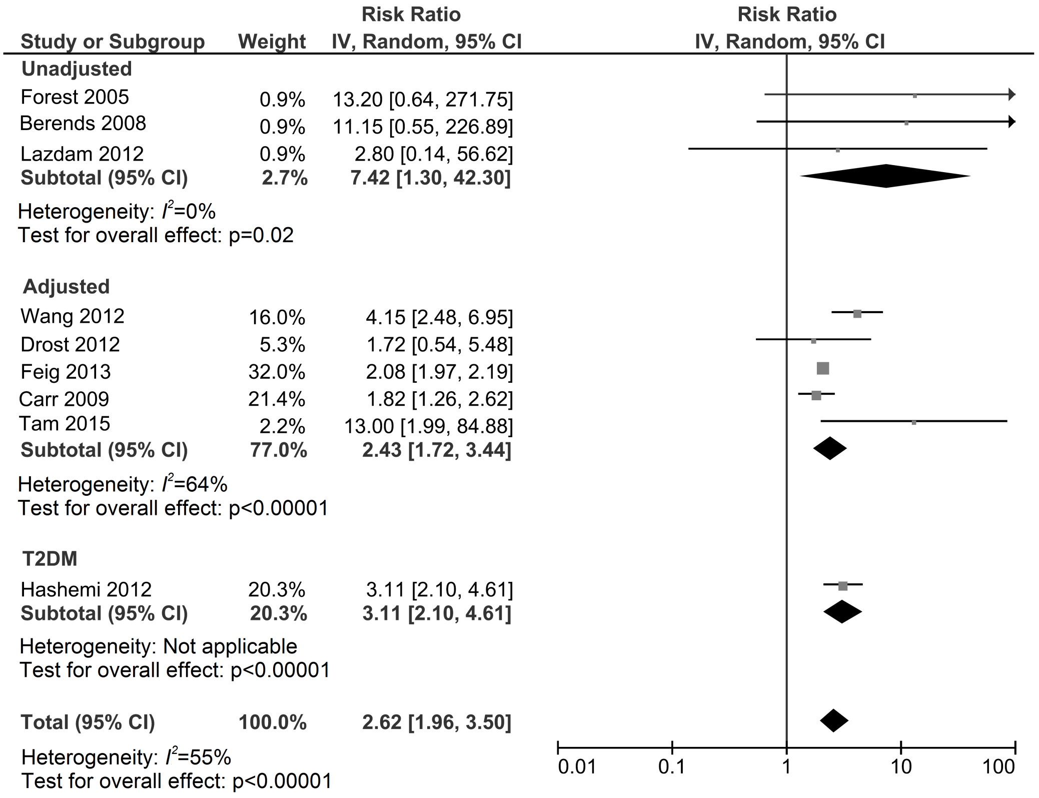

ESM Figure 3. Sensitivity analysis of studies with follow-up 6-10 years.

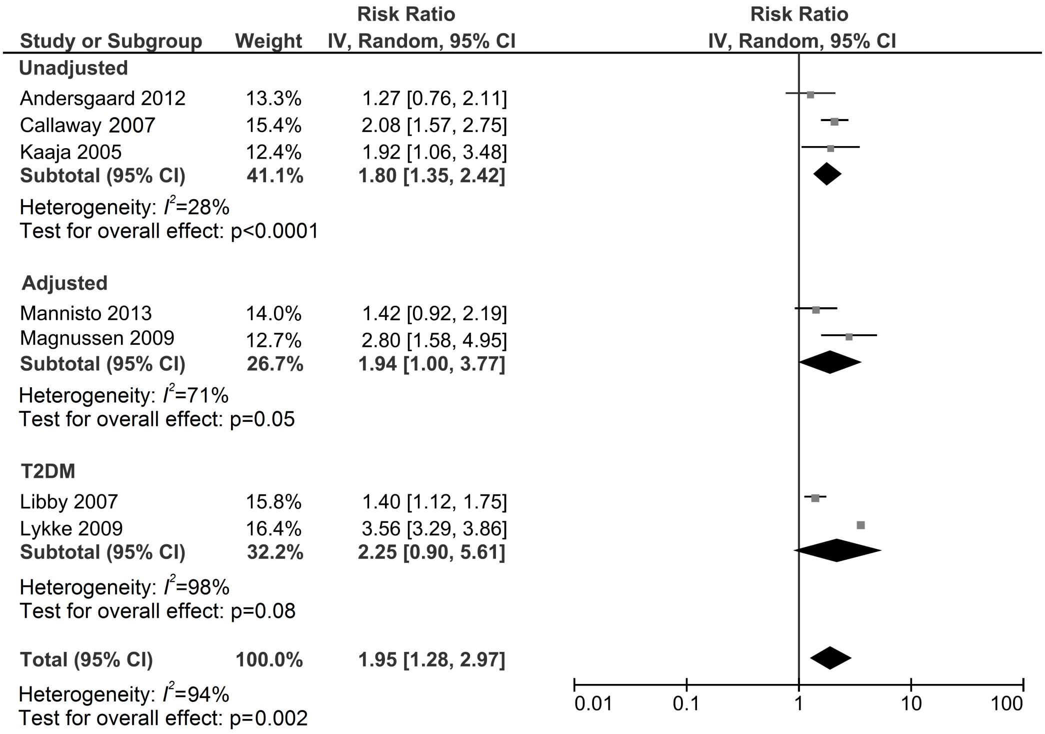

ESM Figure 4. Sensitivity analysis of studies with follow-up >10 years

Study or Subgroup	Weight	Risk Ratio IV, Random, 95% CI
**Unadjusted**		
Callaway 2007	9.7%	2.08 [1.57, 2.75]
Forest 2005	0.5%	13.20 [0.64, 271.75]
Berends 2008	0.5%	11.15 [0.55, 226.89]
Lazdam 2012	0.5%	2.80 [0.14, 56.62]
Breetveld 2014	0.5%	1.32 [0.05, 33.30]
**Subtotal (95% CI)**	**11.8%**	**2.14 [1.62, 2.82]**
Heterogeneity: I^2=0%		
Test for overall effect: p<0.00001		
**Adjusted**		
Mannisto 2013	8.1%	1.42 [0.92, 2.19]
Wang 2012	7.2%	4.15 [2.48, 6.95]
Feig 2013	11.4%	2.08 [1.97, 2.19]
Carr 2009	8.8%	1.82 [1.26, 2.62]
Engeland 2011	10.5%	3.00 [2.45, 3.67]
Magnussen 2009	6.6%	2.80 [1.58, 4.95]
Tam 2015	1.3%	13.00 [1.99, 84.88]
**Subtotal (95% CI)**	**53.9%**	**2.41 [1.88, 3.09]**
Heterogeneity: I^2=78%		
Test for overall effect: p<0.00001		
**T1DM**		
Savitz 2014	4.9%	1.80 [0.83, 3.92]
**Subtotal (95% CI)**	**4.9%**	**1.80 [0.83, 3.92]**
Heterogeneity: Not applicable		
Test for overall effect: p=0.14		
**T2DM**		
Libby 2007	10.3%	1.40 [1.12, 1.75]
Lykke 2009	11.3%	3.56 [3.29, 3.86]
Savitz 2014	7.9%	2.00 [1.27, 3.14]
**Subtotal (95% CI)**	**29.5%**	**2.17 [1.08, 4.37]**
Heterogeneity: I^2=97%		
Test for overall effect: p=0.03		
**Total (95% CI)**	**100.0%**	**2.34 [1.86, 2.93]**
Heterogeneity: I^2=91%		
Test for overall effect: p<0.00001		

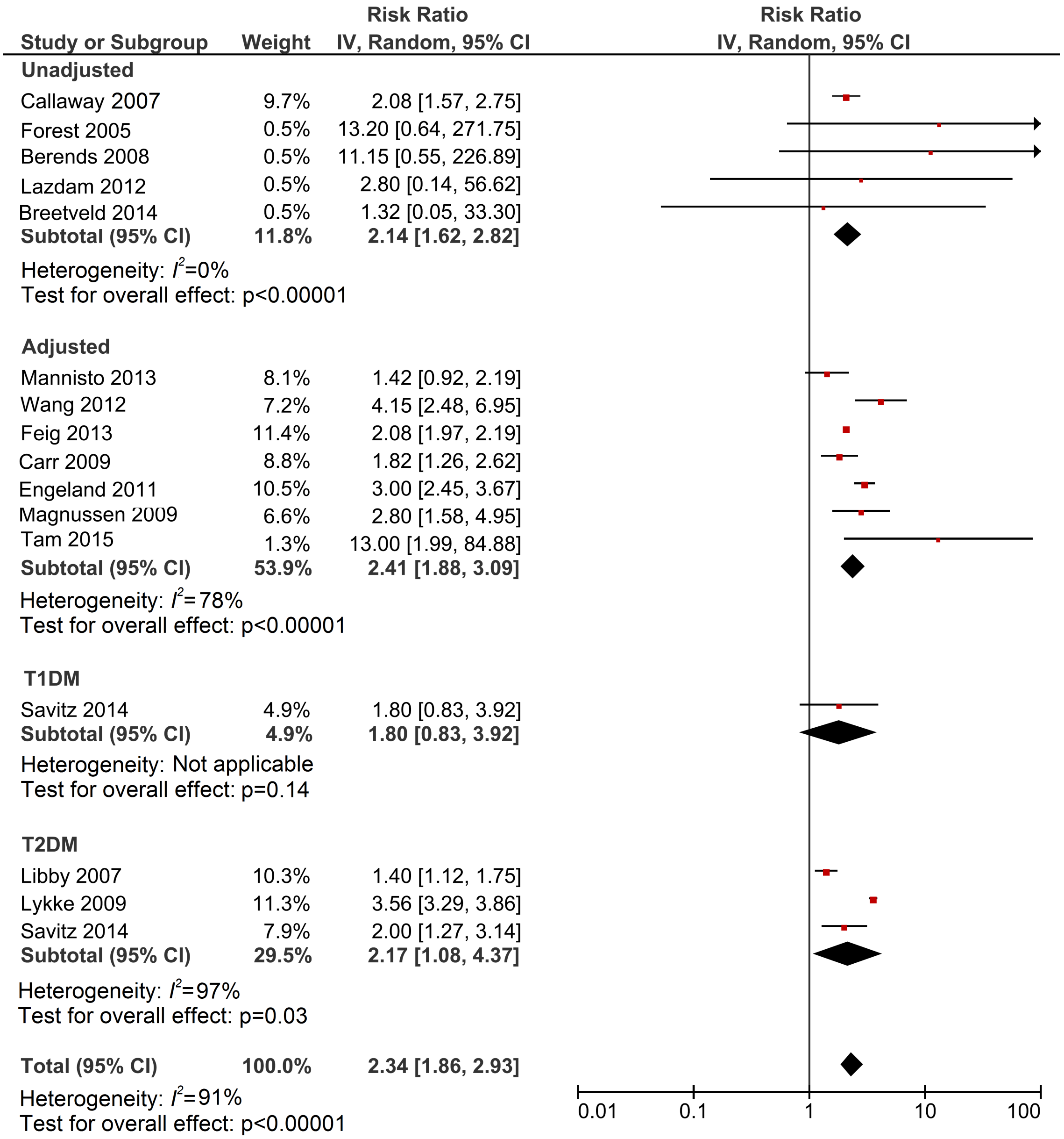

ESM Figure 5. Sensitivity analysis excluding baseline diabetes

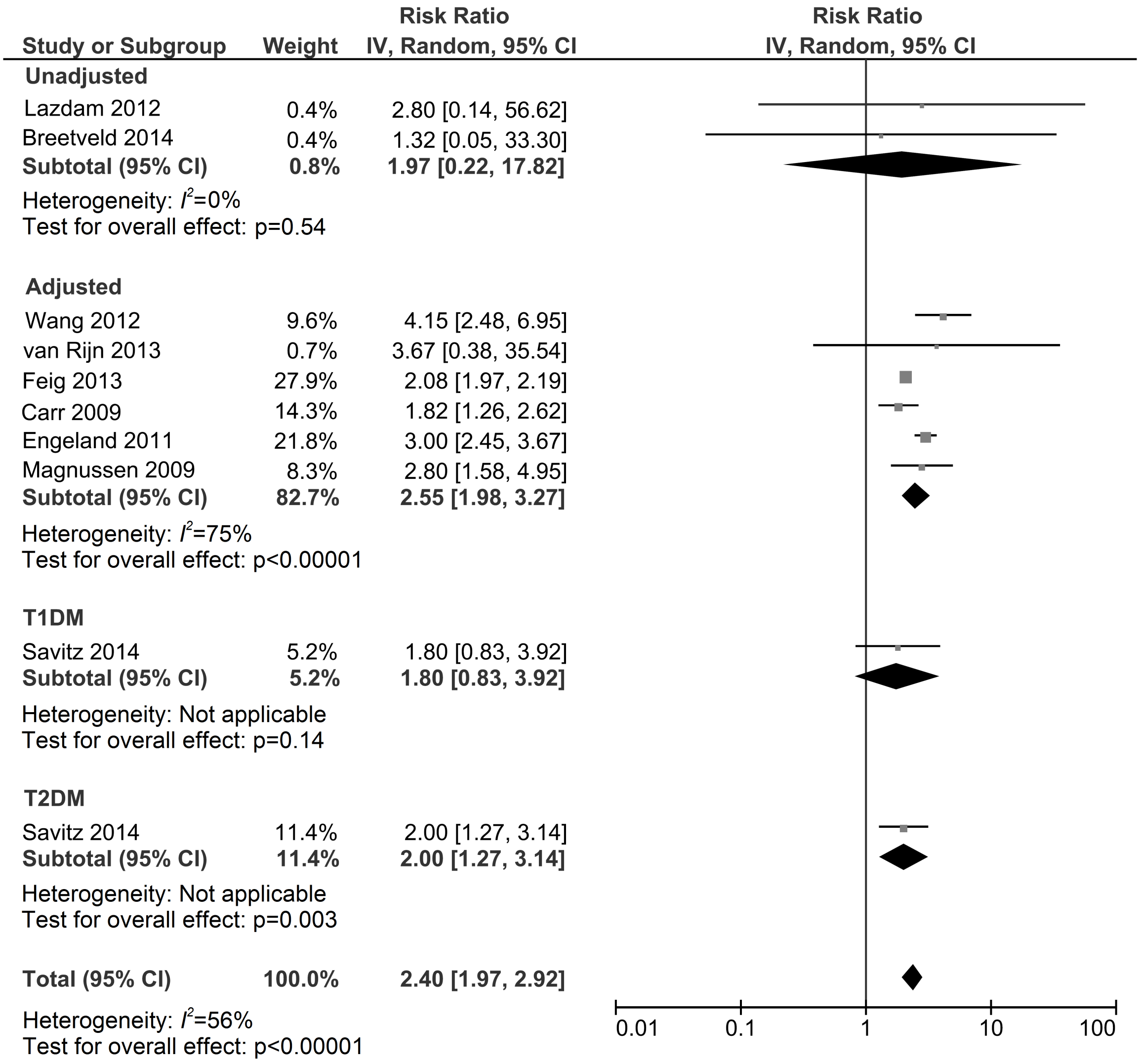

ESM Figure 6. Sensitivity analysis excluding baseline hypertension

Study or Subgroup	Weight	Risk Ratio IV, Random, 95% CI
**Adjusted**		
Mannisto 2013	18.1%	1.42 [0.92, 2.19]
Edlow 2009	4.8%	1.84 [0.51, 6.63]
Wang 2012	15.8%	4.15 [2.48, 6.95]
Magnussen 2009	14.4%	2.80 [1.58, 4.95]
**Subtotal (95% CI)**	**53.0%**	**2.41 [1.37, 4.24]**
Heterogeneity: I^2=71%		
Test for overall effect: p=0.002		
**T1DM**		
Savitz 2014	10.1%	1.80 [0.83, 3.92]
**Subtotal (95% CI)**	**10.1%**	**1.80 [0.83, 3.92]**
Heterogeneity: Not applicable		
Test for overall effect: p=0.14		
**T2DM**		
Hashemi 2012	19.3%	3.11 [2.10, 4.61]
Savitz 2014	17.6%	2.00 [1.27, 3.14]
**Subtotal (95% CI)**	**36.9%**	**2.53 [1.64, 3.90]**
Heterogeneity: I^2=52%		
Test for overall effect: p<0.0001		
**Total (95% CI)**	**100.0%**	**2.38 [1.74, 3.24]**
Heterogeneity: I^2=55%		
Test for overall effect: p<0.00001		

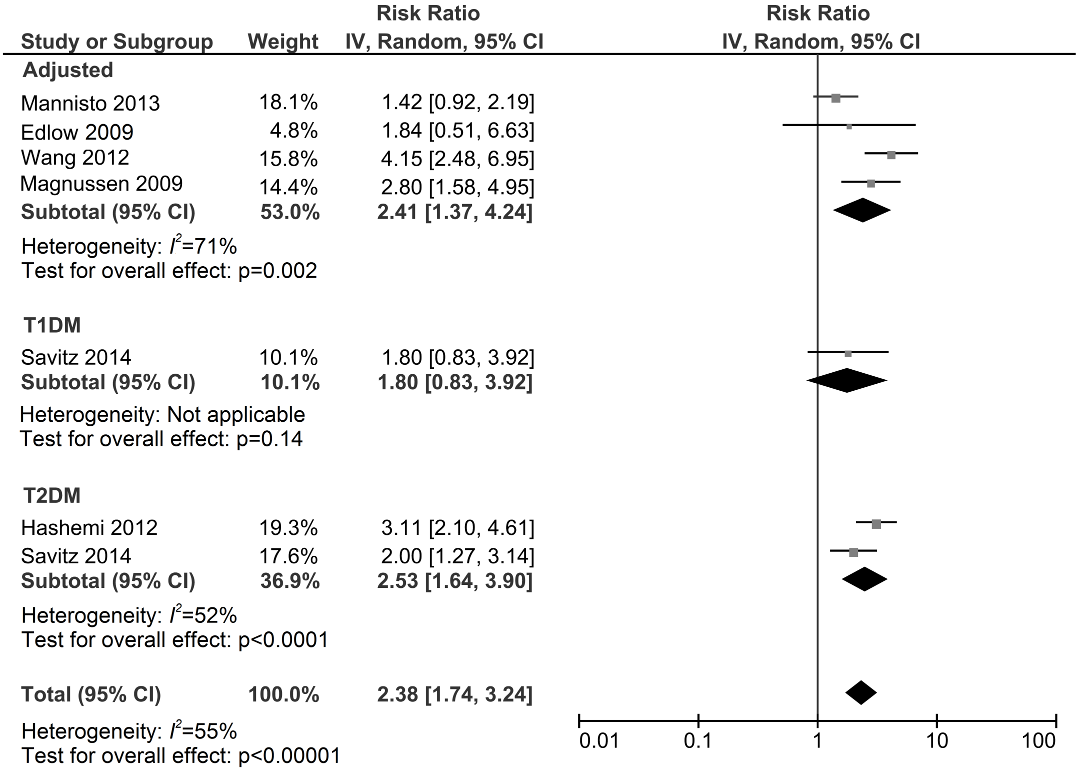

ESM Figure 7. Sensitivity analysis of studies which adjusted for BMI.

Study or Subgroup	Weight	Risk Ratio IV, Random, 95% CI
**Adjusted**		
Wang 2012	27.5%	4.15 [2.48, 6.95]
Magnussen 2009	24.7%	2.80 [1.58, 4.95]
**Subtotal (95% CI)**	**52.2%**	**3.48 [2.37, 5.10]**
Heterogeneity: I^2=1%		
Test for overall effect: p<0.00001		
**T1DM**		
Savitz 2014	16.5%	1.80 [0.83, 3.92]
**Subtotal (95% CI)**	**16.5%**	**1.80 [0.83, 3.92]**
Heterogeneity: Not applicable		
Test for overall effect: p=0.14		
**T2DM**		
Savitz 2014	31.3%	2.00 [1.27, 3.14]
**Subtotal (95% CI)**	**31.3%**	**2.00 [1.27, 3.14]**
Heterogeneity: Not applicable		
Test for overall effect: p=0.003		
**Total (95% CI)**	**100.0%**	**2.61 [1.79, 3.80]**
Heterogeneity: I^2=44%		
Test for overall effect: p<0.00001		

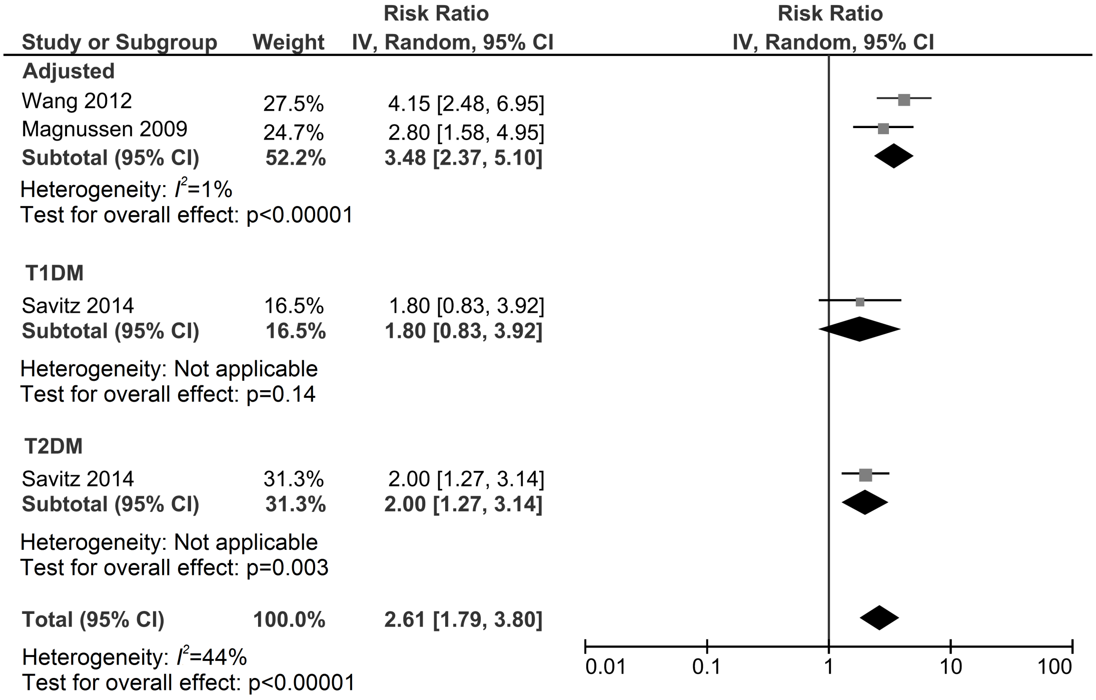

ESM Figure 8. Sensitivity analysis of studies which adjusted for BMI excluding baseline hypertension and diabetes.

Study or Subgroup	Weight	Risk Ratio IV, Random, 95% CI
**Matched**		
Forest 2005	0.5%	13.20 [0.64, 271.28]
Lazdam 2012	0.5%	2.80 [0.14, 57.25]
**Subtotal (95% CI)**	**1.1%**	**6.07 [0.72, 51.38]**
Heterogeneity: I^2=0%		
Test for overall effect: p=0.10		
**Adjusted**		
Mannisto 2013	8.1%	1.42 [0.92, 2.19]
Wang 2012	7.2%	4.15 [2.48, 6.95]
Drost 2012	2.9%	1.72 [0.54, 5.48]
van Rijn 2013	0.9%	3.67 [0.38, 35.64]
Feig 2013	11.3%	2.08 [1.97, 2.19]
Carr 2009	8.8%	1.82 [1.26, 2.62]
Engeland 2011	10.5%	3.00 [2.45, 3.67]
Magnussen 2009	6.7%	2.80 [1.58, 4.95]
**Subtotal (95% CI)**	**56.3%**	**2.32 [1.85, 2.91]**
Heterogeneity: I^2=70%		
Test for overall effect: p<0.00001		
**T1DM**		
Savitz 2014	4.9%	1.80 [0.83, 3.92]
**Subtotal (95% CI)**	**4.9%**	**1.80 [0.83, 3.92]**
Heterogeneity: Not applicable		
Test for overall effect: p=0.14		
**T2DM**		
Hashemi 2012	8.5%	3.11 [2.10, 4.61]
Libby 2007	10.3%	1.40 [1.12, 1.75]
Lykke 2009	11.2%	3.56 [3.29, 3.86]
Savitz 2014	7.7%	2.00 [1.25, 3.20]
**Subtotal (95% CI)**	**37.7%**	**2.37 [1.37, 4.10]**
Heterogeneity: I^2=95%		
Test for overall effect: p=0.002		
**Total (95% CI)**	**100.0%**	**2.35 [1.87, 2.95]**
Heterogeneity: I^2=92%		
Test for overall effect: p<0.00001		

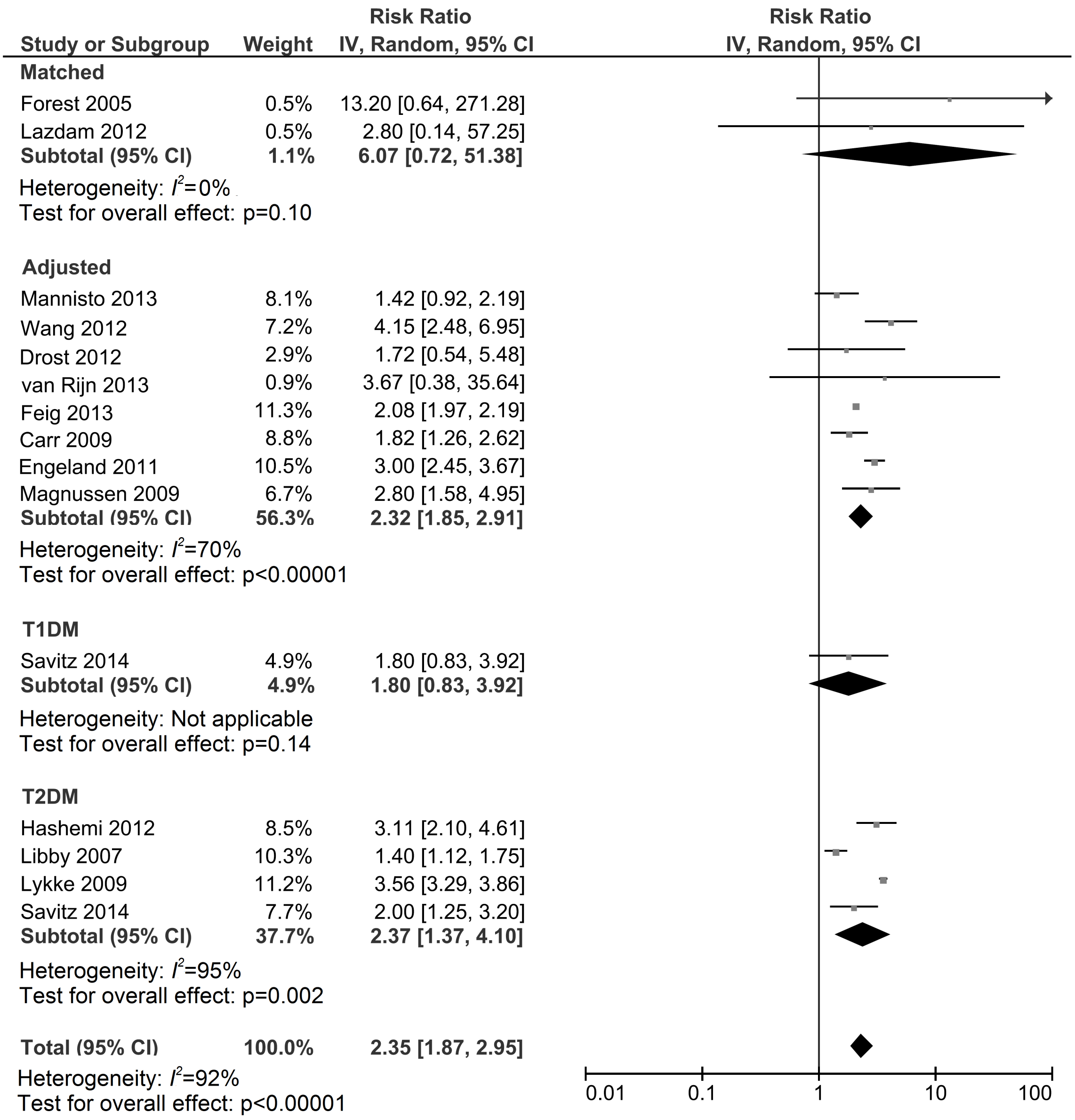

ESM Figure 9. Sensitivity analysis of studies which adjusted for age.

Study or Subgroup	Weight	Risk Ratio IV, Random, 95% CI
**Excluded GDM unadjusted**		
Callaway 2007	16.5%	2.08 [1.57, 2.75]
**Subtotal (95% CI)**	**16.5%**	**2.08 [1.57, 2.75]**
Heterogeneity: Not applicable		
Test for overall effect: p<0.00001		
**Excluded GDM adjusted**		
Wang 2012	9.2%	4.15 [2.48, 6.95]
Feig 2013	24.3%	2.08 [1.97, 2.19]
Engeland 2011	19.6%	3.00 [2.45, 3.67]
Tam 2015	1.1%	13.00 [2.08, 81.09]
**Subtotal (95% CI)**	**54.3%**	**2.96 [2.04, 4.29]**
Heterogeneity: I^2=86%		
Test for overall effect: p<0.00001		
**Adjusted for GDM**		
Carr 2009	13.4%	1.82 [1.26, 2.62]
**Subtotal (95% CI)**	**13.4%**	**1.82 [1.26, 2.62]**
Heterogeneity: Not applicable		
Test for overall effect: p=0.001		
**Adjusted for GDM T1DM**		
Savitz 2014	5.5%	1.80 [0.85, 3.80]
**Subtotal (95% CI)**	**5.5%**	**1.80 [0.85, 3.80]**
Heterogeneity: Not applicable		
Test for overall effect: p=0.12		
**Adjusted for GDM T2DM**		
Savitz 2014	10.3%	2.00 [1.25, 3.20]
**Subtotal (95% CI)**	**10.3%**	**2.00 [1.25, 3.20]**
Heterogeneity: Not applicable		
Test for overall effect: p=0.004		
**Total (95% CI)**	**100.0%**	**2.36 [1.94, 2.88]**
Heterogeneity: I^2= 69%		
Test for overall effect: p<0.00001		

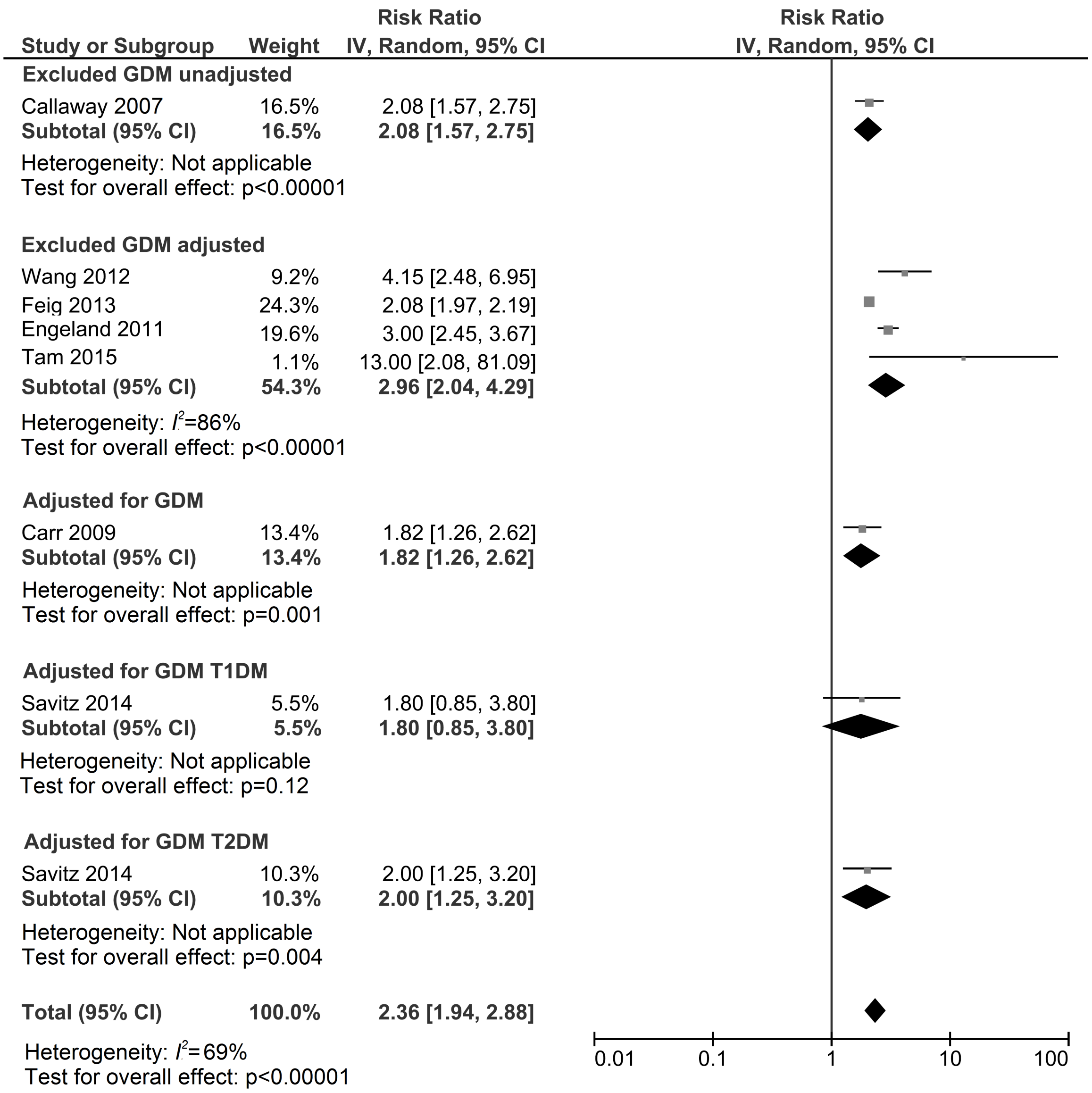

ESM Figure 10. Sensitivity analysis of studies which excluded or adjusted for gestational diabetes.
